# Supplementary material for: Mental health service delivery among adolescent girls and young women (AGYW) seeking HIV prevention and treatment services in central Kenya: A qualitative study of AGYW and healthcare providers’ perceptions
Source: PLoS One. 2025 Dec 5;20(12):e0337795. doi: 10.1371/journal.pone.0337795 (PMC12680144; doi:10.1371/journal.pone.0337795)
Supplement: S1 File — SRQ-20-item mental health screening tool. (PDF) [file pone.0337795.s001.pdf]

Self-Reporting Questionnaire 20-Item Mental Health Screening Tool

**Symptoms of common mental disorders**<br>***The following questions are related to certain pains and problems that may have bothered you in the last 30 days. If you think the question applies to you and you had to describe the problem in the last 30 days, answer YES. On the other hand, if the question does not apply to you and you did not have the problem in the last 30 days, answer NO.***		
1.	Do you often have headaches?	☐ *Yes*<br>☐ *No*
2.	Is your appetite poor?	☐ *Yes*<br>☐ *No*
3.	Do you sleep badly?	☐ *Yes*<br>☐ *No*
4.	Are you easily frightened?	☐ *Yes*<br>☐ *No*
5.	Do your hand shake?	☐ *Yes*<br>☐ *No*
6.	Do you feel nervous, tense, or worried?	☐ *Yes*<br>☐ *No*
7.	Is your digestion poor?	☐ *Yes*<br>☐ *No*
8.	Do you have trouble thinking clearly?	☐ *Yes*<br>☐ *No*
9.	Do you feel unhappy	☐ *Yes*<br>☐ *No*
10.	Do you cry more than usual?	☐ *Yes*<br>☐ *No*
11.	Do you find it difficult to enjoy your daily activities?	☐ *Yes*<br>☐ *No*
12.	Do you find it difficult to make decisions?	☐ *Yes*<br>☐ *No*
13.	Is your daily work suffering?	☐ *Yes*<br>☐ *No*
14.	Are you unable to play a useful part in life?	☐ *Yes*<br>☐ *No*
15.	Have you lost interest in things?	☐ *Yes*<br>☐ *No*
16.	Do you feel that you are a worthless person?	☐ *Yes*<br>☐ *No*
17.	Has the thought of ending your life been on your mind?	☐ *Yes*<br>☐ *No*
18.	Do you feel tired all the time?	☐ *Yes*<br>☐ *No*
19.	Do you have uncomfortable feelings in your stomach?	☐ *Yes*<br>☐ *No*
20.	Are you easily tired?	☐ *Yes*<br>☐ *No*
