# Supplementary material for: Mental health service delivery among adolescent girls and young women (AGYW) seeking HIV prevention and treatment services in central Kenya: A qualitative study of AGYW and healthcare providers’ perceptions
Source: PLoS One. 2025 Dec 5;20(12):e0337795. doi: 10.1371/journal.pone.0337795 (PMC12680144; doi:10.1371/journal.pone.0337795)
Supplement: S2 File — AGYW interview guide. (PDF) [file pone.0337795.s002.pdf]

Appendix IV. In-depth Interview Topic Guide – AGYW

Title: Developing a simulated patient encounter intervention to improve provider training around screening, counseling, and referring for common mental disorders among adolescents in an HIV clinic in Thika, Kenya

Protocol Version 1.2
Date 14 October 2020

Interviewer instructions: Administer informed consent. Once signed, begin guide.

**0.0 Interview Information**<br>***Fill out items A through F prior to starting interview.***
(a) Informed consent has been administered: YES / NO<br>*If consent form has not been signed by participant, interview must not proceed.*<br><br>(b) Interview ID: ___________________________<br>*Format: Interviewee Type-DDMMYY-Number interview conducted that day where "U" = PrEP user and "P" = peer of PrEP users (i.e., PrEP non-user) (e.g., "U-021220-02" represents the second PrEP user interviewed on 12 Feb 2020)*<br><br>(c) Date of interview: ____/_____/_______<br>*Format: DD/MM/YYYY*<br><br>(d) Location of interview: _______________________________________<br><br>(e) Interviewer's full name: ______________________________________<br><br>(f) Interview start time: ___________________<br>*Format: HH:MM am or pm*<br><br>(g) Interview end time: ____________________<br>*Format: HH:MM am or pm*

Facilitator introduction: [DO NOT READ; GUIDE ONLY]

Hello. My name is _______, and I am a ________, working at _______. Thank you for taking the time to talk with me today.

The purpose of this interview is to understand your experiences with symptoms of mental health issues and your feelings about receiving mental health services in HIV clinics as part of your HIV treatment or prevention services.

There are no right or wrong answers to these questions. People have different views and we are interested to learn more about these experiences from you. Today, you are in the role of a teacher and I am here to learn from you since you are an expert in your own life experiences and opinions.

This interview should take around an hour to complete. Please let me know if at any time you have questions, if something I say is not clear, or if you need to take a break. Before we start, do you have any questions?

Part 1. Basic information about the participant
As I mentioned, the goal of this work is to develop a model for mental healthcare that can fit in with HIV service delivery. I'd like to start today by just getting to know you and your experiences with HIV services a bit.<br><br>***1.1 Can you tell me what your experience is with visiting this HIV clinic?***<br><br>• How long have you been visiting this HIV clinic for services? How often do you come to this clinic? Have you visited other clinics for similar services?<br>• What services do you receive from HIV clinics (e.g., antiretroviral therapy, pre-exposure prophylaxis, HIV testing, other)?<br>• Describe how these visits go for you (e.g., how long do they take?, do you always see the same provider?)<br><br>***1.2 How satisfied do you feel with the quality of the HIV treatment or prevention you receive?***<br><br>• What would you change?<br>• What would you not change?<br>• Do you feel comfortable talking with your HIV provider(s) about your sexual behavior and other issues affecting your health? Why or why not?
**Part 2. Experiences of symptoms of mental health issues**
Now I'd like to transition to talking more about mental health. Symptoms of mental health issues are very common among young women around your age and can include feelings of depression, stress, anxiety or worry. I would like to hear about your opinions on mental health symptoms in general and then talk about any mental health symptoms you have experienced recently.<br><br>***2.1 We'd like to understand more about how mental health issues are discussed for young women in Kenya. Can you tell me the specific words that might be used to describe symptoms of "depression"? What about "anxiety", "stress", or "worry"?***<br><br>• Can you describe what someone might look like when they are feeling depressed? How might they behave?<br>• Can you describe what someone might look like when they are feeling anxious, stressed, or worried? How might they behave?<br>• Are there any other mental health issues that are common to young women you know? How are they described? How are they experienced? What are their symptoms?<br><br>***2.2 Describe to me any experiences you've had with symptoms of mental health issues***<br><br>• What words would you use to describe these symptoms?<br>• When did these symptoms occur?

- How severe were the symptoms?
- How long did they last?
- How did you act when you were experiencing the symptoms?
- How did they affect you, your relationships, and your life?

2.3 What about your friends? How common is it for young women around your age to experience symptoms of mental health issues?

- What stories have you heard about mental health issues among your peers?
- When people in your community talk about mental health, how do mental health symptoms come up?
- What did they actually say? Can you cast your mind back to an instance when you heard somebody mention a mental health issue, and remember the conversation?
- Was there ever a time when you noticed a friend or other young woman around your age was experiencing a mental health issue? How did that person act? Did they talk to you about it?

2.4 Describe to me any experiences you or your friends have had with getting screening or treatment for a mental health issue.

- Did you visit a healthcare provider to talk about your mental health symptoms? Why or why not?
- If yes, please describe that discussion with the provider. What did you tell the provider? How did he or she respond? Can you remember details of the conversation and words that you each used?
- Have any of your peers visited a healthcare provider to talk about their mental health symptoms? Why or why not?
- If yes, please describe what you know about that discussion with the provider. What did your friend tell the provider? How did he or she respond? Can you remember any details from your friend's story?

2.5 What would you tell a friend who was experiencing symptoms of a mental health issue?

- Would you recommend the friend seek care from a healthcare provider? Why or why not? If yes, where should she seek care? If yes, how should she initiate this conversation with the healthcare provider?

Part 3. Feelings about receiving mental health services in HIV clinics

For this final part of the interview, I'd like to talk about your feelings about receiving mental health services from your HIV providers in the HIV clinic.

3.1 Mental health care could include screening for a mental health condition, counseling, a medication, or treatment in a psychiatric facility. Describe to me different attitudes towards mental health care in your community.

- Describe to me attitudes toward receiving mental health care among your family members, among your peers?

- How do these attitudes differ from your own?
- How do these attitudes differ from individuals in your larger community?

3.2 What (if anything) worries you about receiving mental health care from your HIV provider(s)?

- Do you have any concerns about combining mental health care with HIV services? Tell me more about these.
- Do you think your peers would have any concerns about combining mental health care with HIV services? Tell me more about these.

3.3 What (if anything) excites you about receiving mental health care from your HIV provider(s)?

- Would you like to visit an HIV clinic that is providing screening for mental health issues, counseling, and referrals in the future? Why or why not?

3.4 What advice would you give to an HIV provider who wants to start providing mental health services to young women in an HIV clinic?

- How should the HIV provider start the conversation about mental health with young women?
- What services should they offer? Why?
- How should they talk to young women about mental health issues (e.g., any specific words that they should use)?

We have come to the conclusion of the topics I had prepared to discuss today. Do you have any other thoughts that you would like to share? Do you have any questions from me before we conclude?

THANK YOU FOR YOUR TIME!

[Mark interview end time on page 1 (Item F).]
