# Supplementary material for: Mental health service delivery among adolescent girls and young women (AGYW) seeking HIV prevention and treatment services in central Kenya: A qualitative study of AGYW and healthcare providers’ perceptions
Source: PLoS One. 2025 Dec 5;20(12):e0337795. doi: 10.1371/journal.pone.0337795 (PMC12680144; doi:10.1371/journal.pone.0337795)
Supplement: S3 File — Healthcare provider interview guide. (PDF) [file pone.0337795.s003.pdf]

Appendix V. In-depth Interview Topic Guide – Healthcare Provider

Title: Developing a simulated patient encounter intervention to improve provider training around screening, counseling, and referring for common mental disorders among adolescents in an HIV clinic in Thika, Kenya

Protocol Version 1.2
Date 14 October 2020

Interviewer instructions: Administer informed consent. Once signed, begin guide.

**0.0 Interview Information**<br>***Fill out items A through F prior to starting interview.***
(a) Informed consent has been administered: YES / NO<br>*If consent form has not been signed by participant, interview must not proceed.*<br><br>(b) Interview ID: ______________________________<br>*Format: Interviewee Type-DDMMYY-Number interview conducted that day where "U" = PrEP user and "P" = peer of PrEP users (i.e., PrEP non-user) (e.g., "U-021220-02" represents the second PrEP user interviewed on 12 Feb 2020)*<br><br>(c) Date of interview: ____/_____/_______<br>*Format: DD/MM/YYYY*<br><br>(d) Location of interview: ____<br><br>(e) Interviewer's full name: ____<br><br>(f) Interview start time: ____________________<br>*Format: HH:MM am or pm*<br><br>(g) Interview end time: _____________________<br>*Format: HH:MM am or pm*

Facilitator introduction: [DO NOT READ; GUIDE ONLY]

Hello. My name is _______, and I am a ________, working at _______. Thank you for taking the time to talk with me today.

The purpose of this interview is to understand your experiences with providing mental health services in HIV clinics to date, your perceptions of delivering mental health screening, counseling, and referrals in the future, and any needs for training and supervision to support mental health service delivery by HIV providers.

There are no right or wrong answers to these questions. People have different views and we are

interested to learn more about these experiences from you. Today, you are in the role of a teacher and I am here to learn from you since you are an expert in your own life experiences and opinions.

This interview should take around an hour to complete. Please let me know if at any time you have questions, if something I say is not clear, or if you need to take a break. Before we start, do you have any questions?

**Part 1. Basic information about the participant**
As I mentioned, the goal of this work is to develop a model for mental healthcare that can fit in with HIV service delivery. I'd like to start today by just getting to know you and your experiences with HIV and mental health services a bit.<br><br>***1.1 Can you tell me a bit about your professional life?***<br><br>• Where do you work?<br>• How long have you worked there?<br>• What does a typical day look like for you?<br>• How often do you see young women, between the ages of 16-25, for care? What services do they come to care for? How long do you typically spend with them on an average clinic visit?<br><br>***1.2 Adolescent girls and young women are often a unique population with specific needs around HIV and mental health care. How satisfied do you feel with the quality of the training you have received to date on providing [SELECT BASED ON PROVIDER TYPE: HIV services, mental health care, general health care] for young women?***<br><br>• Do you feel comfortable talking with young women about their sexual behavior, mental health, and other issues affecting their health? Why or why not?<br>• What would you change about the training you have received to date?<br>• What would you not change about the training you have received to date?<br><br>***1.3 Describe a clinic visit with a young woman that went particularly well.***<br><br>• Why do you think it went well?<br>• What did you learn from this patient encounter?<br><br>***1.4 Describe a clinic visit with a young woman that did not go well.***<br><br>• Why do you think it did not go well?<br>• What did you learn from this patient encounter?<br><br>***1.5 What do you think are some of your strengths as a provider? What do you think are some of your weaknesses?***<br><br>• Think specifically about strengths and weaknesses that might be relevant to your interactions with adolescent girls and young women patients.

Part 2. Experiences of providing mental health services

Now I'd like to transition to talking more about mental health. Symptoms of mental health issues are very common among young women between the ages of 16-25 and can include feelings of depression, stress, or worry. I would like to hear about your experiences, if any, providing mental health services for these conditions and your opinions on providing mental health services within HIV clinics in the future.

2.1 We'd like to understand more about how mental health issues are discussed for young women in Kenya. Can you tell me the specific words that might be used to describe symptoms of "depression"? What about "anxiety", "stress", or "worry"?

- Can you describe what a young woman might look like when she is feeling depressed? How might she behave?
- Can you describe what a young woman might look like when she is feeling anxious, stressed, or worried? How might she behave?
- Are there any other mental health issues that are common to young women you have treated? How were they described? How were they experienced? What are their symptoms?

2.2 Describe to me some experiences you've had with screening young women for symptoms of mental health issues, providing counseling, and/or referring young women to further mental health services.

- What experiences come to mind? When did they occur? What mental health services did you provide?
- What prompted you to deliver these services? What did the patient say? How did she appear during her clinic visit?
- How did she respond to the services you provided?
- What went well in these experiences? What did not go well?
- What lessons did you learn from these experiences for the future?
- IF NO SERVICES PROVIDED: What are some reasons why you have not provided mental health services for young women?

Part 3. Perceptions of providing mental health services in the future and needs for training and supervision for providing mental health care in HIV clinics

For this final part of the interview, I'd like to talk about your feelings about providing mental health services in the HIV clinic and your feelings about different training and supervision approaches.

3.1 What concerns do you have about providing screening for symptoms of mental health services, counseling, and/or referrals for young women?

- Do you have any concerns about combining mental health care with HIV services? Tell me more about these.
- Do you think your peers would have any concerns about combining mental health care with HIV services? Tell me more about these.

3.2 What (if anything) excites you about providing mental health care for young women

in HIV clinics?

- Would you like to work at an HIV clinic that is providing screening for mental health issues, counseling, and referrals for young women in the future? Why or why not?

3.3 Describe to me different attitudes towards providing screening for mental health issues, counseling young women for mental health issues, and providing referrals in your clinic environment.

- Describe to me attitudes toward providing mental health care among your peers in the clinic.
- From your supervisor?
- From other employees at the clinic?
- How do these attitudes differ from your own?

3.4 A few years ago, the Kenyan Ministry of Health released a National Mental Health Policy that includes a recommendation for integrated mental health and HIV care. Had you heard anything about this policy?

- IF YES: What had you heard about the policy?
- IF YES: What is your opinion of this policy? What are others' opinions of this policy?
- IF YES: How do you think it could be implemented in your clinic?

3.5 What advice would you give to an HIV provider who wants to start providing mental health services to young women in an HIV clinic?

- How should the HIV provider start the conversation about mental health with young women?
- What services should they offer? Why?
- How should they talk to young women about mental health issues (e.g., any specific words that they should use)?

3.6 Have you ever received training on mental health service delivery? This could include training on how to screen patients for symptoms of mental health issues, how to discuss those issues with patients and provide counseling, and/or how to provide referrals for mental health care.

- Describe the training you have received.
- When did you receive this training? Who provided this training?
- IF PARTICIPANT HAS RECEIVED TRAINING ON A COUNSELING APPROACH: What type of counseling approach have you been trained on? Do you know the name? Could you describe some of the key components of the counseling sessions?

3.7 What additional training or support would you need to provide mental health services to young women in an HIV clinic?

- What are some core components of this training you would like in the future?

- Describe an ideal model of training and supervision that would help you screen young women for mental health issues, provide some counseling at the clinic, and decide when to provide referrals.

We have come to the conclusion of the topics I had prepared to discuss today. Do you have any other thoughts that you would like to share? Do you have any questions from me before we conclude?

THANK YOU FOR YOUR TIME!

[Mark interview end time on page 1 (Item F).]
