# Supplementary material for: Mental health service delivery among adolescent girls and young women (AGYW) seeking HIV prevention and treatment services in central Kenya: A qualitative study of AGYW and healthcare providers’ perceptions
Source: PLoS One. 2025 Dec 5;20(12):e0337795. doi: 10.1371/journal.pone.0337795 (PMC12680144; doi:10.1371/journal.pone.0337795)
Supplement: S4 File — AGYW codebook. (PDF) [file pone.0337795.s004.pdf]

<u>JiTunze AGYW Interview Codebook</u>

Code Name	Code Description
**A. Descriptions of HIV service delivery**	**Apply this code to any description of AGYW experience with visiting HIV clinic and receiving services in the HIV clinic that does not fit with other sub-codes in the "A" group.**
A1. Experience of HIV service	Any mention of AGYW's HIV clinic visit experiences, including types of services received, length of time they have received services, frequency of clinic visits, waiting times, number and types of providers seen during visits, and whether they get similar services from other clinics.
A2. Perception of HIV services	Any mention of AGYW's positive or negative feelings about clinic visits and HIV services received, including good or bad quality of services, things they like or dislike, things they would change or not change, and reasons for likes and dislikes.
A3. Comfort with HIV providers	Any mention of whether AGYW feel comfortable talking with HIV providers, including reasons for comfort and specific narratives of comfortable interactions with providers.
A4. Discomfort with HIV providers	Any mention of whether AGYW feel uncomfortable talking with HIV providers, including reasons for discomfort and specific narratives of uncomfortable interactions with providers.
**B. Knowledge of mental health issues**	**Apply this code to any description of AGYW general knowledge of mental health issues and their symptoms that do not fit with other sub-codes in the "B" category.**
B1. Description of mental health issues	Any mention of AGYW's knowledge and description of common mental health issues affecting AGYW, and words used by AGYW to describe common mental health symptoms like 'stress', 'worry', 'depression' or 'anxiety'.
B2. Symptoms of mental health issues	Any mention of AGYW's description of symptoms of depression, anxiety, stress, worry, or other mental health issues common to AGYW including how people look or behave when experiencing these mental health issues.
**C. Personal experiences of mental health symptoms**	**Apply this code to any description of AGYW, peer and community experiences with symptoms of mental health issues, including mental health stories and conversations that do not fit with other sub-codes in the "C" category.**
C1. Perceived prevalence of AGYW mental health issues in the community	Any mention of whether it is common for AGYW in the community and peers to experience symptoms of mental health issues. Also include description of discussions and conversations in the community concerning mental health issues heard by AGYW, including what is said and words used during such conversations.

C2. AGYW mental health discussions	Any mention of stories told by AGYW about mental health issues, including description of symptoms of mental health issues common to AGYW and their causes. Also include how discussions and conversations about mental health symptoms come up, and specific words used during such conversations.
C3. Peers mental health experiences	Any mention of peers' experiences with mental health issues, including description of issues they experienced, whether they talked to AGYW about the issues, description of mental health symptoms related to the experiences, including how they looked or behaved, words used to describe the symptoms, when symptoms occurred, severity and duration of symptoms, and how symptoms affected peers' life and relationships.
C4. AGYW personal mental health experiences	Any mention of AGYW's recent experience with mental health issues, including description of issues they experienced, mental health symptoms related to the experiences, including how they looked or behaved, words used to describe the symptoms, when symptoms occurred, severity of symptoms, duration of symptoms, and how symptoms affected AGYW's life and relationships..
**D. Descriptions of mental health service delivery**	**Apply this code to any description of AGYW and peers' experiences of receiving mental health services from a healthcare provider that do not fit with other sub-codes in the "D" category.**
D1. AGYW mental health service experience	Any mention of whether AGYW have received mental health services from a healthcare provider, their experience of receiving the services, mental health symptoms discussed with the provider, and words used during the discussions with provider. Also include AGYW's feelings while talking to mental health providers, whether they felt comfortable or uncomfortable, and reasons for comfort or discomfort while receiving services.
D2. Peer's experience of mental health services	Any mention of whether peers have received mental health services from a healthcare provider, their experience of receiving the services, mental health symptoms discussed with the provider, and words used during the discussion with provider. Also include peers' feelings while talking to mental health providers, whether they felt comfortable or uncomfortable, and their response to the discussion with provider.
D3. Facilitators and barriers to seeking mental health services	Any mention of AGYW or peers' reasons for visiting or not visiting a mental healthcare provider. Also include any mention of whether AGYW know where to refer peers to seek mental healthcare, and their advice to peers on how to initiate a conversation with a mental healthcare provider.
**E. Community attitudes towards mental health services**	**Apply this code to descriptions of attitudes of AGYW, community, family and peers about mental health issues and receiving mental health services from healthcare providers, including what AGYW, community, family and peers say or think about mental health issues, and comparison of attitudes between these groups, including reasons for**

	**differences. Also include AGYW's advice to peers experiencing mental health issues, and whether AGYW would recommend mental healthcare to peers, with reasons.**
**F. Acceptability of receiving mental health services in HIV clinics**	**Apply this code to descriptions of AGYW and peers' concerns and preferences about receiving mental healthcare from providers in HIV clinics, including reasons for the concerns and preferences. Also include any mention of things AGYW and peers like or dislike about receiving mental health services from HIV clinics with reasons, and whether AGYW would be willing or not to receive mental health services in HIV clinics in future, with reasons.**
**G. Recommendations to HIV providers**	**Apply this code to any recommendations to HIV providers for mental health service delivery to AGYW in HIV clinics.**
G1. Provider preferences	Any mention of AGYW's advice to HIV providers who want to start providing mental health services to AGYW in a HIV clinic. Also include suggestions of services HIV providers should provide to AGYW in HIV clinics.
G2. Provider approach for counseling messaging	Any mention of how HIV providers should start a conversation about mental health with young women. Also include any mention of how providers should talk to AGYW about mental health issues, including preferred language and specific words.
**H. Other suggestions**	Any mention of other suggestions related to mental health services not included in G.
**I. Exemplary quotes**	**Apply this code to any outstanding quotes that occur throughout the transcripts to be used in the final analysis.**
