# Supplementary material for: Mental health service delivery among adolescent girls and young women (AGYW) seeking HIV prevention and treatment services in central Kenya: A qualitative study of AGYW and healthcare providers’ perceptions
Source: PLoS One. 2025 Dec 5;20(12):e0337795. doi: 10.1371/journal.pone.0337795 (PMC12680144; doi:10.1371/journal.pone.0337795)
Supplement: S5 File — Healthcare provider codebook. (PDF) [file pone.0337795.s005.pdf]

JiTunze Provider Interview Codebook

Code Name	Code Description
**A. General service delivery experiences**	**Apply this code to any description of provider's professional information and general experiences with providing HIV or mental health services to AGYW that does not fit with other sub-codes in the "A" group.**
A1. Professional information	Any mention of provider's professional information, including department, role and length of time working in HIV or mental health clinic, and how their typical day looks like.
A2. Experience with AGYW services	Any mention of provider's experience with seeing AGYW for care, how often they see AGYW for care, types of services AGYW seek, and length of time spent with AGYW during a clinic visit. Also include any mention of provider's comfort with providing services to AGYW, including reasons for comfort or discomfort.
A3. Quality of training	Any mention of whether provider is satisfied with the quality of training they have received in relation to providing healthcare service to AGYW, including what they would change and what they would not change about the training, with reasons.
A4. Positive clinic encounter with AGYW	Any mention of provider's positive clinic encounter with AGYW, including reasons why the encounter was positive and lessons learnt from the encounter.
A5. Negative clinic encounter with AGYW	Any mention of provider's negative clinic encounter with AGYW, including reasons why the encounter was negative and lessons learnt from the encounter.
A6. Provider strengths and weaknesses	Any mention of things the provider finds easy to do or strengths, and the things they find challenging to do or weaknesses, including how these influence the provider's interactions with AGYW patients.
**B. Mental health service knowledge**	**Apply this code to any description of provider's knowledge and experiences with mental health issues affecting AGYW that does not fit with other sub-codes in the "B" group.**
B1. Description of mental health issues	Any mention of provider's knowledge and description of common mental health issues affecting AGYW, including words used by AGYW to describe common mental health symptoms like 'stress', 'worry', 'depression' or 'anxiety'.
B2. Symptoms of mental health issues	Any mention of provider's description of symptoms of depression, anxiety, stress, worry and other mental health issues common to AGYW, including how AGYW look or behave when experiencing these mental health issues.
**C. Mental health service delivery experiences**	**Apply this code to any description of provider's experience with providing mental health services (screening, counseling and/or referrals for further mental health services) to AGYW that does not fit with other sub-codes in the "C" group.**

C1. Example mental health service delivery	Any mention of provider's description of an outstanding mental health service they have delivered to AGYW, when they occurred, and the mental health services the provider offered. Also include the considerations that prompted provider to offer mental health services to AGYW, what the AGYW said, how AGYW appeared during the clinic visit, and the response of the AGYW to the services provided
C2. Perception of service delivery experience	Any mention of things that went well or did not go well when providing mental health services to AGYW, including reasons. Also include lessons learnt from the mental health service delivery experience to inform future service delivery to AGYW.
C3. Barriers to mental health service delivery	Any mention of whether provider has not provided mental health services for AGYW, including reasons for these.
**D. Acceptability of mental health service delivery within HIV clinics**	**Apply this code to any description of provider's feelings about providing mental health services for AGYW within HIV clinics that does not fit with other sub-codes in the "D" group.**
D1. Provider concerns	Any mention of provider and peer's concerns about providing mental health services (screening, counseling, and referral) for AGYW, and any concerns about combining mental healthcare with HIV services, including reasons for these concerns.
D2. Provider preferences	Any mention of the things provider likes about providing mental healthcare for AGYW in HIV clinics, whether they would like to work in a HIV clinic providing mental health services to AGYW, and the reasons for these preferences. Also include provider's advice to HIV providers who want to start providing mental health services to AGYW in an HIV clinic, how HIV providers should start a conversation about mental health issues with young women, services providers should provide to AGYW in HIV clinics, and how providers should talk to AGYW about mental health issues, including language and specific words.
D3. Provider attitudes	Any mention of provider's, peer's and supervisor's attitudes towards providing mental health services for AGYW within their clinic environment. Also include any comparison of attitudes of provider from those of peers and supervisors, including reasons.
**E. Mental health policy knowledge**	**Any mention of provider's knowledge of Kenya Mental Health policy, including what they know about the policy, their own and peer's opinion of the policy, and suggestions on how the policy could be implemented within their clinic environment.**
**F. Recommendations for mental health service delivery to AGYW in HIV clinics**	**Apply this code to descriptions of provider's advice to HIV providers who want to start providing mental health services to AGYW in HIV clinics, including how to talk to AGYW.**

**G. Training and supervision approaches**	**Apply this code to descriptions of different training and supervision approaches for mental health service delivery.**
G1. Mental health service training	Any mention of whether provider has received a training for providing mental health services to patients, description of the training received, when the training was received and who provided the training. Will often be double coded with code A4.
G2. Counseling approach training	Any mention of whether provider has received a training on a counseling approach, the type and/or name of counseling approach trained on, and description of the key components of the counseling sessions used in the approach. Will often be double coded with code A4.
G3. Training and supervision needs	Any mention of provider's needs for training or support that they would need to provide mental health services to AGYW in future, including the core components of the training. Will often be double coded with code A4, F1, and/or F2.
G4. Ideal training	Any mention of provider's description of an ideal model of training and supervision they require to help them provide screening, counseling and referral for AGYW within their clinic environment.
**H. Other suggestions**	**Apply this code to any mention of other suggestions related to mental health service delivery not included in "F" or "G".**
**I. Exemplary quotes**	**Apply this code to any outstanding quotes that occur throughout the transcripts to be used in the final analysis.**
