# Supplementary material for: Mental health service delivery among adolescent girls and young women (AGYW) seeking HIV prevention and treatment services in central Kenya: A qualitative study of AGYW and healthcare providers’ perceptions
Source: PLoS One. 2025 Dec 5;20(12):e0337795. doi: 10.1371/journal.pone.0337795 (PMC12680144; doi:10.1371/journal.pone.0337795)
Supplement: Attachment — Submitted filename: Review of PONE-D-24-60333.docx [file pone.0337795.s006.docx]

Abstract:

Overall – the abstract needs work to align with the paper better and have consistent language use, it needs to be rewritten. The SRQ-20 sounds so basic, consider adding that it is a mental health screening tool (or even call it MHST or SRQ-20-MHST). In methods you need to more plainly say that you quantified CMDs (versus “explored experiences of CMDs”) and that you analyzed qualitative data versus just analyzed data using content analysis. You are using a priori coding methods along with inductive methods, you clearly have codes you are interested in (eg., access to services, provider attitudes about mental health services, etc.). What does this mean “AGYW described CMD experiences related to multi-level risk factors…”? This is an example of a rewrite needed. What is a clinician compared to a nurse? A nurse is a clinician too, what cadre are you referring to?

**Introduction:**

68-69: You state that AGYW were chosen due to disproportionate burden of HIV and CMDs but these data are not shown, what is their burden compared to the general population?

75: There needs to be a better link between these two sentences.

78: Consider adding quick sentence about this paper’s focus.

83: “more than 10%” makes no sense, replace with actual %

85-90: This is too much information about Kenya without anything earlier stating that the study is set there. Consider deleting 87-90 or integrating it elsewhere. This section should be focused first on SSA then go into Kenya (with exception that you use Kenya as an example with references 19-22)

101: “Despite an abundance of research in SSA focused on HIV in adolescents…”. Also the last sentence here “mental health is still poorly understood and under-prioritized within HIV research and interventions…” needs more emphasis. This needs to come through in abstract and at start of introduction, its very important.

109: “experiences with mental health services among AGYW…”. Why Central, Kenya??

Overall: I don’t see GBV mentioned whatsoever and this burden is twinned almost always with HIV for AGYW, this needs to be integrated into literature search and throughout Introduction. An important link is that if AGYW have suffered GBV they are at risk of further harm from GBV (and the associated mental health issues from both) and are at higher risk of HIV, etc.

**Methods:**

114: This first sentence needs to be rewritten, what is the purpose of the JiTunze study?

Study Population section:

This is a bit of a mish mash. The sampling methods should be its own section. The whole section needs a rewrite to be more plain and simple.

122: What kind of interviews?

123: screening and enrollment *for the study*?

124: PHRD, add if this is a clinic, a hospital, what kind of facility? Tertiary? We can tell what a subcounty hospital may be like but not PHRD.

127: See my notes about renaming SRQ-20 to be less vague.

131: “their capacity to consent”. I’m glad you had this procedure in the study. Do you know how many referrals from the study were actually linked to services?

138: isn’t the sample of providers only coming from within the 2 identified facilities, not any facility as you state?

141: “all interviews with AGYW and providers…” and (author EO)

155: Did you stop the interview or complete later? Did they go for support after the interview was completed?

157: We directly transcribed audio-recorded interviews verbatim regardless of language and translated Kiswahili transcripts into English.

175: consider “multilevel factors” instead of social factors (esp. since that word shows up later in the paper) and also including why this model is good for AGYW (very much affected by circles of influence outside of themselves) and add more literature for why this model fits well for AGYW.

Ethical statement: what did you do with the audio recordings, including phone recordings.

**Results**

Participant characteristics: is there a reason you aren’t including # HIV negative versus # HIV positive? This is relevant I think to the findings presented in the paper. I would add in addition to the number of providers and their role, how many from each facility? And what kind of facility?

AGYW experience of CMD is one section and “Risk factors for CMDs” is another.

Overall: sometimes you say experiences with CMD and sometimes symptoms, which one is best to use and stick with it. You definitely have a priori themes in the results, you need to change methods to say you had a priori codes you were looking for in the data.

215: the fact that you are wanting to find individual level risk factors is not coming through in abstract, introduction or methods.

216: “risk factor for experiencing…”

219: “and fear of potentially transmitting…”

223: Maybe just me but I would want to state the service, like “as one AGYW seeking PrEP reported:”

244: This is very interesting that AGYW are conflating any individual attention given to somehow receiving counseling…this is not commented on in discussion and I think it should be. For one, a simple change in practice is to have more individual attention and less group adherence counseling which occurs in a lot of places. Did you ask more about group versus individual services? I would look for any qualitative data about group versus individual counseling for AGYW getting HIV services and see if you see anything interesting that can be added to the paper. For sure I think group counseling may be an opportunity lost to talk to an individual AGYW about stress, anxiety, depression, GBV, poverty, etc. Generally, this is an excellent section…

261: Interesting that this quote is essentially the definition of resiliency. Maybe comment on that here or in discussion? Also anything to add to discussion about stress? It seems a common complaint but also vague, any literature showing that AGYW or others use this word as an all encompassing word for a mental health concern? I heard it a lot in these kinds of settings too.

273: “delivering mental health services to AGYW than HIV providers did”. “However, HIV care providers’ lower confidence in providing mental health services was mainly due to…”

277: “however, more specific screening tools …”

279: internalized stigma

285: I have never heard this ever that an HIV clinic had support groups for AGYW unless it was an outside program like DREAMS, it just doesn’t happen. I would verify this information with Kenyan colleagues or make a note anywhere you talk about support groups that they were non-governmental additional services brought by an outside group…unless he is totally making this up to make the clinic look good?

297: You just wrote that AGYW get referred. So is this counseling done by the clinic where the AGYW presented or at the referral site? Need to clarify.

306: This is another quote that needs verification I believe. You may just want to find out what % of AGYW (or any patient) is physically escorted. This is so uncommon in these settings and this nurse may not be entirely truthful about all AGYW being physically escorted, this is so uncommon in a very busy clinic that staff can escort patients. Or explain if we are talking about a different department at a hospital??

310: Consider ‘access’ instead of delivery and ‘by AGYW’; check abstract an intro to see if factors influencing access to services is coming through strongly enough …

339: consider, “Conversely, mental healthcare providers…”

344: rewrite for better clarity

349: Don’t need however. What does “difficulty of AGYW gaining insight” mean?

350: mental health stigma?

353-359: I don’t see this quote as showing that some people think mental health care providers behave like their clients, I would delete that conclusion. They are just saying that providers may be afraid of MH patients because they could be aggressive or harm them.

366: “since adolescents had more routine HIV care parameters” what does this mean? Rewrite these sentences here because it is important to note clearly why providers thought it was different to work with adolescents and why exactly.

369: “screening into HIV clinics” or into HIV service delivery

389: Benefit of integrating …?

390-396: needs rewrite for more clarity

409: CMD symptoms, substance use relapses, …

415-431: See notes above about individual versus group counseling, did this come up in the interviews re. how to integrate MH services

Discussion

436: “contrasting service access and delivery gaps among AGYW and providers”. What does this mean? Can be stated in plain language like differences of opinion or something like that.

446-450: This jumps out early in discussion, esp. after just reading those findings the page before. Suggest moving this to join with other service integration language later in Discussion.

473-476: why are they more challenging? I’m still not seeing why. Cite any literature where providers consider serving adolescents as like serving children, they are looked down on as a population to serve.

Overall: it seems that you have chosen to endorse integration over a stand alone youth center, as mentioned by the providers and which is in fact is a very common way to deal with the very real difficulties in integrating adolescent care into an adult setting. This again is a priori coding and should be stated as such, that you were interested in finding out AGYW and providers view on integration…but take a look to see if you can also mention that some providers support youth centers as an alternative and that perhaps future studies should compare and contrast the two models for adolescent mental health, including cost, to determine which model leads to better outcomes.
