# Supplementary material for: Mental health service delivery among adolescent girls and young women (AGYW) seeking HIV prevention and treatment services in central Kenya: A qualitative study of AGYW and healthcare providers’ perceptions
Source: PLoS One. 2025 Dec 5;20(12):e0337795. doi: 10.1371/journal.pone.0337795 (PMC12680144; doi:10.1371/journal.pone.0337795)
Supplement: Attachment — Submitted filename: Response to Reviewers.docx [file pone.0337795.s009.docx]

18^th^ March 2025

The Academic Editor

PLOS ONE

Dear Dr., Akinola,

RE: Response to Manuscript ID PONE-D-24-60333

We thank the reviewers for your review of our manuscript entitled, “**Mental health service delivery among adolescent girls and young women (AGYW) seeking HIV prevention and treatment services in central Kenya: a qualitative study of AGYW and healthcare providers’ perceptions**”, and the opportunity to resubmit a revised version. We appreciate your and the reviewers’ valuable feedback, and we are pleased to incorporate these recommendations. We are resubmitting the revised manuscript that addresses all suggestions. Below is an updated point-by-point response to the comments along with a description of the changes that have been made to address the comments. The reviewer comments are included in bold text, and our responses are standard font. We appreciate, in advance, your time and consideration of this revised manuscript for publication in PLOS ONE.

Sincerely,

Jennifer Velloza (on behalf of all authors)

University of California San Francisco (UCSF)

**Editor Comments:**

**Journal requirements:**

**When submitting your revision, we need you to address these additional requirements.**

**1. Please ensure that your manuscript meets PLOS ONE's style requirements, including those for file naming. The PLOS ONE style templates can be found at**

[**https://journals.plos.org/plosone/s/file?id=wjVg/PLOSOne_formatting_sample_main_body.pdf**](https://journals.plos.org/plosone/s/file?id=wjVg/PLOSOne_formatting_sample_main_body.pdf) **and**

[**https://journals.plos.org/plosone/s/file?id=ba62/PLOSOne_formatting_sample_title_authors_affiliations.pdf**](https://journals.plos.org/plosone/s/file?id=ba62/PLOSOne_formatting_sample_title_authors_affiliations.pdf)

We have made changes throughout the manuscript to meet PLOS ONE’s style requirements, including file naming, layout, headings, citation formatting, and supporting information.

**2. Thank you for stating the following financial disclosure:**

**‘The study was funded by a pilot award from the University of Washington Global Mental Health Program to the senior author (JV).”**

**Please state what role the funders took in the study. If the funders had no role, please state: "The funders had no role in study design, data collection and analysis, decision to publish, or preparation of the manuscript."**

**If this statement is not correct you must amend it as needed.**

**Please include this amended Role of Funder statement in your cover letter; we will change the online submission form on your behalf.**

We have included this funding statement in the cover letter, which now reads: “This study was funded by a pilot award from the University of Washington Global Mental Health Program to the senior author (JV). The funders had no role in the study design, data collection and analysis, decision to publish, or preparation of the manuscript.”

**3. In this instance it seems there may be acceptable restrictions in place that prevent the public sharing of your minimal data. However, in line with our goal of ensuring long-term data availability to all interested researchers, PLOS’ Data Policy states that authors cannot be the sole named individuals responsible for ensuring data access (http://journals.plos.org/plosone/s/data-availability#loc-acceptable-data-sharing-methods).**

**Data requests to a non-author institutional point of contact, such as a data access or ethics committee, helps guarantee long term stability and availability of data. Providing interested researchers with a durable point of contact ensures data will be accessible even if an author changes email addresses, institutions, or becomes unavailable to answer requests.**

**Before we proceed with your manuscript, please also provide non-author contact information (phone/email/hyperlink) for a data access committee, ethics committee, or other institutional body to which data requests may be sent. If no institutional body is available to respond to requests for your minimal data, please consider if there any institutional representatives who did not collaborate in the study, and are not listed as authors on the manuscript, who would be able to hold the data and respond to external requests for data access? If so, please provide their contact information (i.e., email address). Please also provide details on how you will ensure persistent or long-term data storage and availability.**

We have updated the data availability statement and included an institutional contact: the International Clinical Research Center (ICRC) at the University of Washington. Staff at the ICRC were not directly involved in the study and are not listed as co-authors on the manuscript. They, however, have access to the data and can respond to external requests for data. Lines 734-741 now read: “The semi-structured interview guides and codebooks used to collect and analyze the qualitative interviews are included as additional files. De-identified transcripts may be available after consultation with the University of Washington and Kenya Medical Research Institute institutional review boards, as per the ethical approval obtained for this study. The full de-identified transcripts will be available upon author request and under appropriate data-sharing agreements to those who provide a methodologically sound proposal by contacting the International Clinical Research Center at the University of Washington (Email: icrc@uw.edu) or the senior author (Email: jennifer.velloza@ucsf.edu).”

**4. We note that you have indicated that there are restrictions to data sharing for this study. PLOS only allows data to be available upon request if there are legal or ethical restrictions on sharing data publicly. For more information on unacceptable data access restrictions, please see http://journals.plos.org/plosone/s/data-availability#loc-unacceptable-data-access-restrictions.**

**Before we proceed with your manuscript, please address the following prompts:**

**a) If there are ethical or legal restrictions on sharing a de-identified data set, please explain them in detail (e.g., data contain potentially identifying or sensitive patient information, data are owned by a third-party organization, etc.) and who has imposed them (e.g., a Research Ethics Committee or Institutional Review Board, etc.). Please also provide contact information for a data access committee, ethics committee, or other institutional body to which data requests may be sent.**

The interview transcripts contain potentially identifying and sensitive information related to young women’s (including minors’) experiences of HIV risk factors and mental health symptoms that could potentially be harmful if their identity was disclosed. Considerable privacy concerns have been noted regarding the confidentiality of qualitative interviews done with adolescents living with HIV and those engaged in high-risk sexual behaviour (MacDonald et al., 2023: [10.1016/j.jpeds.2023.113589](https://doi.org/10.1016/j.jpeds.2023.113589)). Other researchers (e.g., Chauvette et al., 2019: [https://doi.org/10.1177/1609406918823](https://doi.org/10.1177/1609406918823863); and Lamb et al., 2024: [https://doi.org/10.1177/13591053241237](https://doi.org/10.1177/13591053241237620)) have also highlighted various epistemological, methodological, legal, and ethical concerns regarding open data sharing and secondary analysis of qualitative research. These concerns include privacy and confidentiality and potential harm to participants whose identity may be easily reconstructed, especially in small rural communities such as among the adolescents and young women in our peri-urban study setting.

**b) If there are no restrictions, please upload the minimal anonymized data set necessary to replicate your study findings to a stable, public repository and provide us with the relevant URLs, DOIs, or accession numbers. For a list of recommended repositories, please see**

**https://journals.plos.org/plosone/s/recommended-repositories. You also have the option of uploading the data as Supporting Information files, but we would recommend depositing data directly to a data repository if possible.**

**We will update your Data Availability statement on your behalf to reflect the information you provide.**

We have revised the data availability statement in lines 734-741 which now reads: “The semi-structured interview guides and codebooks used to collect and analyze the qualitative interviews are included as additional files. De-identified transcripts may be available after consultation with the University of Washington and Kenya Medical Research Institute institutional review boards, as per the ethical approvals obtained for this study. The full de-identified transcripts will be available upon author request and under appropriate data-sharing agreements to those who provide a methodologically sound proposal by contacting the International Clinical Research Center at the University of Washington (Email: icrc@uw.edu) or the senior author (Email: jennifer.velloza@ucsf.edu).”

**Additional Editor Comments:**

**109- 110: explored experiences of mental health service, delivery among AGYW receiving HIV services and healthcare providers in Central, Kenya. Kindly redefine your objectives or research questions?**

We have made changes to redefine the objectives in the introduction section. Lines 142-144 now read: “We conducted a qualitative study to understand the perceptions of mental health service access and delivery within HIV clinics among AGYW receiving HIV services and healthcare providers in Central, Kenya.”

**121: Kindly include subtitles i.e under study population, state/mention the area or site of study, and justifying why you have chosen that particular place, is it most prevalent or you simply decided, the reason you gave is not justifiable to suffice?. Preceded by we recruited 20 AGYW……**

We have revised the sub-headings to separate the study design and the study setting and population in the methods section. Lines 157-168 now read: “We conducted the study in Thika, an urban center in Kiambu county in Central Kenya, located approximately 40km from the capital, Nairobi, with a population-level HIV prevalence of 2.7% and a large surrounding peri-urban and rural population [36]. Kiambu county hosts approximately 250,000 AGYW of ages 15-24, who account for over 1,000 new HIV infections annually [37]. The primary site for the study, Thika sub-County Hospital (Level 5), was ideal for the study as it provides HIV prevention and treatment services to a large population of AGYW aged 10-24 (~233 living with HIV; and 9 on PrEP). We also included seven surrounding healthcare facilities to increase the diversity of the study population. These included Partners in Health and Research Development (PHRD), a clinical research site that implements HIV prevention and treatment trials among diverse populations including AGYW, Makongeni dispensary (Level 2), Kiandutu health centre (Level 3), Ruiru sub-County hospital (Level 4), Kiambu County referral hospital (Level 5), Mathari National referral hospital (Level 6), and Kenyatta National hospital (Level 6).”

**121- 122: Clearly explain the description of the sampling strategy, including rationale for the recruitment method, and participant.**

We have clarified and included the rationale for the sampling and recruitment strategy. Lines 171-181 now read: “AGYW were referred for screening and enrollment for the study by HIV care providers from Thika sub-County Hospital and the PHRD clinic. Eligible AGYW were aged 16-25 years and were currently receiving HIV prevention, treatment, or testing services at either clinic at the time of enrollment. HIV care providers at the two facilities were sensitized about the study to support the recruitment of AGYW by identifying potential participants while providing HIV services. Once the providers identified AGYW who fit the eligibility criteria, they informed them about the study and referred those who expressed willingness for study participation to the study staff at the PHRD clinic (approximately 1.5 kilometers distance) for screening and enrolment. This assured AGYW of confidentiality, enhanced their comfort in study participation, and built trust between the AGYW and study staff.”

Lines 198-209 now read: “We used stratified purposive sampling to include AGYW in different age groups (16-18, 19-22, and 23-25); and receiving different HIV prevention and treatment services: PrEP, HIV testing services (HTS), post-exposure prophylaxis (PEP), and antiretroviral therapy (ART), to capture variations in experiences of mental health service delivery among different demographic groups [40]. We also purposively sampled HIV and mental healthcare providers to include different cadres (HIV counsellors, clinical officers, nurses, psychiatrists, and psychologists), healthcare facility representation, service delivery experience, and educational backgrounds (college diploma and university degree). The overall sample size of 20 AGYW and 10 providers was determined by thematic saturation, whereby we stopped interviews once no new data emerged [40].”

**Overall, it’s a great article, kindly incorporate the constructive suggestions from the reviewers and myself, thank you.**

Thank you for this positive feedback.

**Reviewer 1 comments**

**Reviewer #1: Abstract**

**- Please add to methods what the time range for data collection was**

We have included the timeframe in the abstract. Lines 40-42 now read: “Between 16th February and 14th June 2021, we conducted in-depth interviews with AGYW receiving HIV services and healthcare providers from eight clinics in Central Kenya.”

**- In results, please clarify if systematic or systemic lack of access to MH services was meant by authors.**

We have revised the sentence in lines 56-57, which now reads: “AGYW reported a high need for mental health services but described a systemic lack of access.”

**- Results seem to switch back and forth between AGYW and providers. Please reorganize and/or clarify how socioecological model informs the structure of the results.**

We have reorganized the results in the abstract, and lines 54-64 now read: “AGYW described experiences of CMDs attributed to individual, interpersonal, and community-level risk factors including HIV stigma, financial, and relationship challenges. AGYW reported a high need for mental health services but described a systemic lack of access. Convenience and positive experiences with providers facilitated AGYW’s access to services. Conversely, HIV care providers felt less confident in delivering mental health services due to inadequate mental health training compared to mental healthcare providers. Providers also reported inadequate training, poor referral systems, and unclear guidelines that hindered service delivery. AGYW and providers endorsed mental health service integration within HIV clinics to potentially reduce referral burden for AGYW and improve service quality.”

**- Results around providers feeling less trained compared to mental health providers is unclear that mental health providers were included in the sample of interviews.**

We have clarified the methods in the abstract to include mental healthcare providers. Lines 51-53 now read: “The providers (n=10) comprised seven females, and included six HIV and four mental healthcare providers.”

**Introduction**

**Overall, excellent introduction to the availability of MH services in Kenya and the overlaps between HIV and CMDs.**

Thank you for this positive feedback.

**- Line 92 unclear if “inadequate mental healthcare providers” is referring to availability of providers or the training is inadequate. Please clarify**

We have revised the sentence in lines 111-114 to now read: “Numerous barriers hinder mental health service delivery among adolescents receiving HIV services in SSA, including stigma, shortage of mental healthcare providers, poor treatment and referral pathways, and a lack of routine screening for CMDs [13,15,16].”

**Methods**

**Thorough explanation of the methodology employed for this study, and gives a good sense of what was covered in the interviews and why those were of interest based on the introduction.**

Thank you for this positive feedback.

**- Line 121 – what kind of providers are included in this category? Please add more detail as to the educational background to qualify for interviews.**

We have revised the methods section to include the provider cadres and educational background. Lines 202-205 now read: “We also purposively sampled HIV and mental healthcare providers to include different cadres (HIV counsellors, clinical officers, nurses, psychiatrists, and psychologists), healthcare facility representation, service delivery experience, and educational backgrounds (college diploma and university degree).”

We have also revised the results to include providers’ educational backgrounds, cadres, and facility representation. Lines 285-293 now read: “The providers included six HIV and four mental healthcare providers (two HIV counselors, three clinical officers [physician assistant equivalent], three nurses, one psychologist, and one psychiatrist). Providers had a median age of 37 years (IQR: 30-44), included 70% (n=7) females, had an average experience of 10 years in HIV or mental health service delivery, and their educational backgrounds included diploma (n=6), higher diploma (n=2) and degree (n=2). The providers represented seven HIV and mental healthcare facilities: Thika sub-County hospital (n=4), and (n=1) each from Makongeni dispensary, Kiandutu health centre, Ruiru sub-County hospital, Kiambu County hospital, Mathari National hospital, and Kenyatta National hospital.”

**Results**

**Quite a lot of themes to cover in this section, but overall great work in explaining the depth of information available in these interviews.**

Thank you for this positive feedback.

**- Please add a summary of themes to better orient readers to the framework and organization of the results.**

We have included a summary of the key themes in the data management and analysis section. Lines 260-266 now read: “We presented our findings of the key themes in the subsequent sections, organized within the different levels of the socio-ecological model (individual, interpersonal, community, provider, service, and structural-level factors). The key themes included: AGYW experiences and symptoms of CMDs, risk factors for CMDs among AGYW, experiences of mental health service delivery within HIV clinics, factors influencing mental health service access and delivery among AGYW, and benefits of integrating mental health services within HIV clinics.”

**- Line 197 unclear how AGYW experiences and risk factors for CMD were a separate theme from Individual-level risk factors. Or whether they were an introduction to the risk factors. Please add language to delineate.**

We have revised the subheading in the results section to separate the two themes. Line 296 now reads: “AGYW experiences and symptoms of CMDs”; line 317 now reads: “Risk factors for CMDs among AGYW.”

**Discussion**

**Well-written contextualization of their data within existing knowledge base.**

Thank you for this positive feedback.

**- Line 455, These results really stands out as a reader. Are these biases against working with adolescents unique to this subset of providers or are there other studies that reflect this reluctance?**

We have included a discussion on provider biases against working with AGYW. Lines 637-656 now read: “In our study, providers’ reluctance to provide services to AGYW due to perceptions that AGYW were ‘difficult clients’, hindered mental health service delivery to AGYW. HIV and mental healthcare providers also perceived delivering mental health services to AGYW to be more challenging and time-consuming compared to other populations, and as ‘extra work’. A recent study among HIV care providers in Kenya, South Africa, and Zimbabwe reported challenges in delivering PrEP services to adolescent girls younger than 18 years, whom providers described as ‘troublesome’, ‘not listening’, and ‘dishonest’ [69]. Other studies have reported beliefs among providers regarding the perceived difficulty of providing care to adolescents with CMDs [53], and the burden from additional screening tools [7]. Studies in SSA have also reported biases among SRH and HIV care providers regarding providing contraception and HIV prevention to AGYW due to cultural stigma towards pre-marital sexual activity among youth [70,71]. In Kenya, low confidence to deliver HIV services to adolescents has been attributed to insufficient provider training on adolescent-friendly HIV care, while access to trained providers and youth-friendly services have been linked to better HIV outcomes [72,73]. In our study, HIV care providers employed youth-friendly strategies like ‘referral by escort’ but lacked specific skills for addressing CMDs and for AGYW-friendly communication and counseling. These findings could explain the providers’ reluctance to deliver mental health services to AGYW, highlighting the need for prioritizing the provision of regular provider training on mental health services and adolescent-friendly care.”

**Reviewer 2 comments:**

**Abstract:**

**Overall – the abstract needs work to align with the paper better and have consistent language use, it needs to be rewritten. The SRQ-20 sounds so basic, consider adding that it is a mental health screening tool (or even call it MHST or SRQ-20-MHST). In methods you need to more plainly say that you quantified CMDs (versus “explored experiences of CMDs”) and that you analyzed qualitative data versus just analyzed data using content analysis. You are using a priori coding methods along with inductive methods, you clearly have codes you are interested in (eg., access to services, provider attitudes about mental health services, etc.). What does this mean “AGYW described CMD experiences related to multi-level risk factors…”? This is an example of a rewrite needed. What is a clinician compared to a nurse? A nurse is a clinician too, what cadre are you referring to?**

We have revised the abstract to align with the paper. Since this was a fully qualitative study, we did not quantify CMDs except for screening purposes. However, we qualitatively explored AGYW’s experiences with CMDs during in-depth interviews.

Line 37-38 now reads: “We conducted a qualitative study to explore AGYW and healthcare providers’ perceptions of mental health service delivery within HIV clinics.”

Line 40-42 now reads: “Between 16th February and 14^th^ June 2021, we conducted in-depth interviews with AGYW receiving HIV services and healthcare providers from eight clinics in Central Kenya.”

Line 42-44 now reads: “Eligible AGYW were aged 16-25 years and reported mild-to-moderate CMD symptoms, determined by the Self-Reporting Questionnaire 20-item (SRQ-20) mental health screening tool.”

Line 51-53 now reads: “The providers (n=10) comprised seven females, and included six HIV and four mental healthcare providers.”

Line 54-56 now reads: “AGYW described experiences of CMDs attributed to individual, interpersonal, and community-level risk factors including HIV stigma, financial, and relationship challenges.”

We have also clarified the language for the SRQ-20-item screening tool and consistently used the terms ‘Self-Reporting Questionnaire 20-item mental health screening tool’, ‘SRQ-20 screening tool’, or SRQ-20 screening score’ throughout the manuscript. Since the SRQ-20 is a widely used and validated self-reporting questionnaire developed by the World Health Organization for screening for common mental disorders in primary healthcare settings, particularly in low- and middle-income countries (e.g., van der Westhuizen et al., 2016; doi: 10.1007/s11469-015-9566-x, and Do et al., 2023; doi: 10.2147/IJWH.S404993), it is well known in the mental health field by the abbreviation ‘SRQ-20’.

**Introduction:**

**68-69: You state that AGYW were chosen due to disproportionate burden of HIV and CMDs but these data are not shown, what is their burden compared to the general population?**

We have made changes to include the CMD prevalence among AGYW. Lines 83-85 now read: “Studies in SSA have reported a CMD prevalence ranging between 30-50% among AGYW receiving HIV services, compared to 10-37% among the general population [9–12].”

**75: There needs to be a better link between these two sentences.**

We have made changes to the sentence and lines 85-88 now read: “CMDs undermine HIV outcomes among AGYW by contributing to low service uptake and retention, poor adherence, limited engagement and persistence in care, and increased sexual risk behaviors, exacerbating AGYW’s risk of HIV acquisition and transmission [13–15].”

**78: Consider adding quick sentence about this paper’s focus.**

We have included a sentence on delivery of mental health services within HIV clinics, and lines 95-96 now read: “Integrating mental health services within HIV clinics could improve access to mental healthcare among vulnerable AGYW populations [7].”

**83: “more than 10%” makes no sense, replace with actual %**

We have revised lines 78-80 to now read: “In Kenya, a recent study among 2106 AGYW aged 12-19 years found that 13 percent had ongoing depression symptoms and one in five had experienced physical or sexual violence in the past 12 months [5].”

**85-90: This is too much information about Kenya without anything earlier stating that the study is set there. Consider deleting 87-90 or integrating it elsewhere. This section should be focused first on SSA then go into Kenya (with exception that you use Kenya as an example with references 19-22)**

We have made changes to introduce Kenya earlier in the introduction section. Lines 78-80 now read: “In Kenya, a recent study among 2106 AGYW aged 12-19 years found that 13 percent had ongoing depression symptoms and one in five had experienced physical or sexual violence in the past 12 months [5].”

We have moved the details about mental health services in Kenya to the end of the paragraph. Lines 116-123 now read: “In Kenya, there is a large gap in mental health service access and delivery within primary healthcare facilities, particularly among adolescents; with low rates of screening and referrals for CMDs within HIV clinics [23–26]. Therefore, Kenyan AGYW with CMDs typically access mental health services such as counseling, psychotherapy, and rehabilitation, at outpatient and inpatient mental health clinics located in sub-county, county, and national-level referral facilities, directly or through referrals by healthcare providers, teachers, and caregivers [23,27].”

**101: “Despite an abundance of research in SSA focused on HIV in adolescents…”. Also the last sentence here “mental health is still poorly understood and under-prioritized within HIV research and interventions…” needs more emphasis. This needs to come through in abstract and at start of introduction, its very important.**

We have moved this sentence to the first paragraph in the introduction section. Lines 92-94 now read: “Despite an abundance of research in SSA focused on HIV in adolescents, mental health is still poorly understood and under-prioritized within HIV research and interventions targeting AGYW [16,17].”

We have revised the abstract, and lines 34-35 now read: “However, mental health is under-prioritized within HIV interventions targeting AGYW.”

**109: “experiences with mental health services among AGYW…”. Why Central, Kenya??**

We have elaborated the justification for the study site in the methods section. Lines 157-160 now read: “We conducted the study in Thika, an urban center in Kiambu county in Central Kenya, located approximately 40km from the capital, Nairobi, with a population-level HIV prevalence of 2.7% and a large surrounding peri-urban and rural population [36]. Kiambu county hosts approximately 250,000 AGYW of ages 15-24, who account for over 1,000 new HIV infections annually [37].”

**Overall: I don’t see GBV mentioned whatsoever and this burden is twinned almost always with HIV for AGYW, this needs to be integrated into literature search and throughout Introduction. An important link is that if AGYW have suffered GBV they are at risk of further harm from GBV (and the associated mental health issues from both) and are at higher risk of HIV, etc.**

We have included gender-based violence in the introduction, results, and discussion sections. In some cases, we have instead used the terms ‘sexual and physical violence’ and ‘intimate partner violence’ as risk factors for CMDs.

Lines 78-80 read: “In Kenya, a recent study among 2106 AGYW aged 12-19 years found that 13 percent had ongoing depression symptoms and one in five had experienced physical or sexual violence in the past 12 months [5].”

Lines 98-102 read: “AGYW in Kenya and other SSA countries have overlapping vulnerabilities to HIV and CMDs due to multi-level risk factors across individual, interpersonal, community, and structural levels. Previous studies have identified key risk factors including physical and sexual violence, intimate partner violence, unfavorable sexual power relations, financial constraints, and low access to sexual and reproductive health (SRH) services [18–20].”

In the results section, lines 344-346 read: “Among AGYW without HIV, mental health symptoms were mostly related to relationship problems including emotional and physical abuse, mistreatment and violence by partners and caregivers, conflicts and separations among parents, sexual violence…”

Lines 357-359 read: “A few AGYW had received mental health counseling related to gender-based violence, unintended pregnancy, relationship challenges with partners, and HIV stigma from providers based at healthcare facilities within their communities.”

Lines 386-389 now read: “Among both HIV and mental healthcare providers, mental health counseling provided to AGYW was mostly related to the reduction of internalized HIV and mental health stigma, social issues like abuse, gender-based violence, and conflicts among AGYW and their caregivers or partners…”

Lines 568-570 now read: “Key CMD risk factors included HIV stigma among AGYW living with HIV; and financial constraints, SRH-related issues, and gender-based violence among AGYW without HIV.”

**Methods:**

**114: This first sentence needs to be rewritten, what is the purpose of the JiTunze study?**

We have made changes to include the purpose of the JiTunze study. Lines 148-152 now read: “This was a formative qualitative study conducted as part of the larger JiTunze pilot study (funded by the University of Washington Global Mental Health Program). This qualitative study aimed to inform the development of a simulated patient encounters (SPE) implementation strategy applied for training HIV care providers to improve mental health service delivery within Kenyan HIV clinics.”

**Study Population section:**

**This is a bit of a mish mash. The sampling methods should be its own section. The whole section needs a rewrite to be more plain and simple.**

We have rewritten the study population section and simplified the language. We have created a heading for the study setting and population in lines 156-168, with separate sub-headings for participant recruitment in lines 169-196, and participant sampling in lines 197-209.

Lines 157-168 now read: “Study setting and population

We conducted the study in Thika, an urban center in Kiambu county in Central Kenya, located approximately 40km from the capital, Nairobi, with a population-level HIV prevalence of 2.7% and a large surrounding peri-urban and rural population [36]. Kiambu county hosts approximately 250,000 AGYW of ages 15-24, who account for over 1,000 new HIV infections annually [37]. The primary site for the study, Thika sub-County Hospital (Level 5), was ideal for the study as it provides HIV prevention and treatment services to a large population of AGYW aged 10-24 (~233 living with HIV; and 9 on PrEP). We also included seven surrounding healthcare facilities to increase the diversity of the study population. These included Partners in Health and Research Development (PHRD), a clinical research site that implements HIV prevention and treatment trials among diverse populations including AGYW, Makongeni dispensary (Level 2), Kiandutu health centre (Level 3), Ruiru sub-County hospital (Level 4), Kiambu County referral hospital (Level 5), Mathari National referral hospital (Level 6), and Kenyatta National hospital (Level 6).”

Lines 169-196 now read: “Participant recruitment

We recruited 20 AGYW and 10 healthcare providers for participation in qualitative in-depth interviews between 16th February and 14th June 2021. AGYW were referred for screening and enrollment for the study by HIV care providers from Thika sub-County Hospital and the PHRD clinic. Eligible AGYW were aged 16-25 years and were currently receiving HIV prevention, treatment, or testing services at either clinic at the time of enrollment. HIV care providers at the two facilities were sensitized about the study to support the recruitment of AGYW by identifying potential participants while providing HIV services. Once the providers identified AGYW who fit the eligibility criteria, they informed them about the study and referred those who expressed willingness for study participation to the study staff at the PHRD clinic (approximately 1.5 kilometers distance) for screening and enrollment. This assured AGYW of confidentiality, enhanced their comfort in study participation, and built trust between the AGYW and study staff.

Once AGYW arrived at the PHRD clinic, they were screened for study participation by a study psychosocial counselor using the Self Reporting Questionnaire 20-item (SRQ-20) mental health screening tool [38], to assess for elevated symptoms of CMDs. AGYW with mild-to-moderate CMD symptoms (determined by a score of 7-14 on the SRQ-20 screening tool), were eligible for interviews. AGYW with scores ≥14, indicating severe CMD symptoms, were excluded from interviews due to potential impacts on capacity to consent for study participation, and risk of harm related to emotional distress from discussing sensitive topics or traumatic experiences [39], and were referred to a study clinical psychologist for psychosocial support.

We recruited healthcare providers from seven HIV and mental healthcare facilities. The providers were eligible if they were aged ≥18 years and had a current role in providing HIV or mental health services in an HIV, mental health, or primary healthcare facility located around Thika and Nairobi areas. The providers were recruited physically during in-person facility visits or using phone calls through which they were invited for study participation.”

Lines 197-209 now read: “Participant sampling

We used stratified purposive sampling to include AGYW in different age groups (16-18, 19-22, and 23-25); and receiving different HIV prevention and treatment services: PrEP, HIV testing services (HTS), post-exposure prophylaxis (PEP), and antiretroviral therapy (ART), to capture variations in experiences of mental health service delivery among different demographic groups [40]. We also purposively sampled HIV and mental healthcare providers to include different cadres (HIV counsellors, clinical officers, nurses, psychiatrists, and psychologists), healthcare facility representation, service delivery experience, and educational backgrounds (college diploma and university degree). The overall sample size of 20 AGYW and 10 providers was determined by thematic saturation, whereby we stopped interviews once no new data emerged [40].”

**122: What kind of interviews?**

We have made the change to “qualitative in-depth interviews”, and lines 170-171 now read: “We recruited 20 AGYW and 10 healthcare providers for participation in qualitative in-depth interviews between 16th February and 14th June 2021.”

**123: screening and enrollment for the study?**

We have made the change, and lines 171-174 now read: “AGYW were referred for screening and enrollment for the study by HIV care providers from Thika sub-County Hospital and the PHRD clinic.”

**124: PHRD, add if this is a clinic, a hospital, what kind of facility? Tertiary? We can tell what a subcounty hospital may be like but not PHRD.**

The PHRD clinic is an HIV clinical trials research site. We have clarified this in the study setting and population section, and lines 163-166 now read: “We also included seven surrounding healthcare facilities to increase the diversity of the study population. These included Partners in Health and Research Development (PHRD), a clinical research site that implements HIV prevention and treatment trials among diverse populations including AGYW…”

**127: See my notes about renaming SRQ-20 to be less vague.**

We have revised the language of the SRQ-20 throughout the manuscript and consistently used the terms ‘Self-Reporting Questionnaire 20-item mental health screening tool’, ‘SRQ-20 screening tool’, or ‘SRQ-20 screening score’ and lines 183-187 now read: “Once AGYW arrived at the PHRD clinic, they were screened for study participation by a study psychosocial counselor using the Self Reporting Questionnaire 20-item (SRQ-20) mental health screening tool [38], to assess for elevated symptoms of CMDs. AGYW with mild-to-moderate CMD symptoms (determined by a score of 7-14 on the SRQ-20 screening tool), were eligible for interviews.”

The SRQ-20 is a widely used and validated self-reporting questionnaire developed by the World Health Organization for screening for common mental disorders in primary healthcare settings, particularly in low- and middle-income countries (e.g., van der Westhuizen et al., 2016; doi: 10.1007/s11469-015-9566-x, and Do et al., 2023; doi: 10.2147/IJWH.S404993), hence it is well known in the mental health field by the abbreviation ‘SRQ-20’.

**131: “their capacity to consent”. I’m glad you had this procedure in the study. Do you know how many referrals from the study were actually linked to services?**

We did not access this data as part of the analysis in the qualitative study component since these AGYW were screened out on entry before enrollment for the in-depth interviews. However, we referred 2-3 AGYW who had shown signs of mental health distress during the interviews to a psychosocial counsellor for support.

**138: isn’t the sample of providers only coming from within the 2 identified facilities, not any facility as you state?**

Healthcare providers were recruited from a total of 7 facilities, while AGYW were recruited from only 2 facilities. We have clarified this in the participant recruitment and results sections.

Lines 192-196 now read: “We recruited healthcare providers from seven HIV and mental healthcare facilities. The providers were eligible if they were aged ≥18 years and had a current role in providing HIV or mental health services in an HIV, mental health, or primary healthcare facility located around Thika and Nairobi areas.”

Lines 290-293 now read: “The providers represented seven HIV and mental healthcare facilities: Thika sub-County hospital (n=4), and (n=1) each from Makongeni dispensary, Kiandutu health centre, Ruiru sub-County hospital, Kiambu County hospital, Mathari National hospital, and Kenyatta National hospital.”

**141: “all interviews with AGYW and providers…” and (author EO)**

We have revised the sentence, and lines 211-214 now read: “All in-depth interviews with AGYW and providers were conducted between 16th February and 14th June 2021 by a trained female qualitative researcher (EO) in-person at the PHRD clinic for AGYW, and at the respective facilities or by phone for providers.”

**155: Did you stop the interview or complete later? Did they go for support after the interview was completed?**

We have clarified the sentence, and lines 226-229 now read: “We paused the interviews if any AGYW showed severe symptoms of mental health distress until they felt comfortable enough to continue with the interviews. We referred these AGYW to a psychosocial counselor at the PHRD clinic for psychosocial support after the interviews were completed.”

**157: We directly transcribed audio-recorded interviews verbatim regardless of language and translated Kiswahili transcripts into English.**

We have made this change, and lines 231-232 now read: “We directly transcribed audio-recorded interviews verbatim regardless of language and translated Kiswahili transcripts into English.”

**175: consider “multilevel factors” instead of social factors (esp. since that word shows up later in the paper) and also including why this model is good for AGYW (very much affected by circles of influence outside of themselves) and add more literature for why this model fits well for AGYW.**

We have made the suggested change and included a description of the suitability of the model for the study. Lines 252-253 now read: “This model has been adapted within public and mental health research to explain the multi-level factors influencing individuals’ health behaviors and risk factors…”

Lines 255-259 now read: “The socio-ecological model was relevant for our study as it provided a comprehensive understanding of the factors influencing AGYW’s mental health, due to the recognition that adolescents and young people’s (AYP) social environments, including their individual experiences, family and peer relationships, and community contexts (e.g., school, and neighborhood) significantly influence their health behaviors and mental health outcomes [46,47].”

**Ethical statement: what did you do with the audio recordings, including phone recordings.**

We have revised the section, and lines 276-278 now read: “All interview and consent audio recordings were stored in a password-protected computer in separate folders and then archived in a secured external hard drive pending deletion 3 years after completion of data analysis and study closure.”

**Results**

**Participant characteristics: is there a reason you aren’t including # HIV negative versus # HIV positive? This is relevant I think to the findings presented in the paper. I would add in addition to the number of providers and their role, how many from each facility? And what kind of facility?**

We have revised the section to include the HIV status of AGYW and the number of providers per facility. We have also provided further descriptions of provider roles.

Lines 283-285 now read: “The AGYW included those living with HIV (n=7), and those without HIV (n=13); with services received including HIV treatment (n=7), PrEP (n=7), and HIV testing and/or PEP (n=6).”

Lines 285-287 now read: “The providers included six HIV and four mental healthcare providers (two HIV counselors, three clinical officers [physician assistant equivalent], three nurses, one psychologist, and one psychiatrist).”

Lines 290-293 now read: “The providers represented seven HIV and mental healthcare facilities: Thika sub-County hospital (n=4), and (n=1) each from Makongeni dispensary, Kiandutu health centre, Ruiru sub-County hospital, Kiambu County hospital, Mathari National hospital, and Kenyatta National hospital.”

**AGYW experience of CMD is one section and “Risk factors for CMDs” is another.**

We have separated the two headings. Line 296 now reads: “AGYW experiences and symptoms of CMDs; and line 316 now reads: “Risk factors for CMDs among AGYW.”

**Overall: sometimes you say experiences with CMD and sometimes symptoms, which one is best to use and stick with it. You definitely have a priori themes in the results, you need to change methods to say you had a priori codes you were looking for in the data.**

We have revised the section to mainly use CMD ‘symptoms’ and only used ‘experiences’ to describe how CMDs were described and felt. Lines 298-306now read: “AGYW commonly described any experiences of CMDs as ‘stress’ and used words in English, Kiswahili, or ‘Sheng’ (an urban slang popular among Kenyan youth) languages, to describe their mental health symptoms. AGYW used words like: ‘niko na stress’ (Sheng- I have stress), ‘kufikiria sana’ (Kiswahili- thinking a lot), ‘kitu inanisumbua’ (Kiswahili- something is disturbing me), ‘mawazo mengi’ (Kiswahili- many thoughts), ‘sifeel poa’ (Sheng- I don’t feel fine), and ‘siko sawa’ (Kiswahili- I am not okay). AGYW also described CMDs using words reflecting self-value perceptions, like ‘I feel useless’ or ‘I feel hopeless’. There were some differences in descriptions of CMD symptoms relative to AGYW’s SRQ-20 screening scores.”

We have revised the methods section to clarify data analysis methods to include both inductive and deductive methods. Lines 232-240 now read: “We analyzed interview data deductively and inductively using thematic content analysis [41], to identify AGYW and providers’ views on factors influencing mental health service access and delivery within HIV clinics. All analyses were performed on de-identified data using Dedoose version 9.0.18 (SocioCultural Research Consultants, LLC, Los Angeles, USA) [42]. First, two authors (EO and JV) reviewed all transcripts, generated key themes, and developed separate codebooks for AGYW and providers using a priori codes from the interview topic guides. The authors iteratively revised the codebooks based on emerging themes from the interview transcripts, then selected a subset (10%; n=3) of AGYW and provider interviews and double-coded them to ensure consistency.”

Lines 260-266 now read: “We presented our findings of the key themes in the subsequent sections, categorized within the different levels of the socio-ecological model (individual, interpersonal, community, provider, service, and structural-level factors). The key themes included: AGYW experiences and symptoms of CMDs, risk factors for CMDs among AGYW, experiences of mental health service delivery within HIV clinics, factors influencing mental health service access and delivery among AGYW, and benefits of integrating mental health services within HIV clinics.”

**215: the fact that you are wanting to find individual level risk factors is not coming through in abstract, introduction or methods.**

We have revised the abstract, introduction, and methods to include the individual-level risk factors. Lines 54-56 now read: “AGYW described experiences of CMDs attributed to individual, interpersonal, and community-level risk factors including HIV stigma, financial, and relationship challenges.”

Lines 98-99 now read: “AGYW in Kenya and other SSA countries have overlapping vulnerabilities to HIV and CMDs due to multi-level risk factors across individual, interpersonal, community, and structural levels.”

Lines 252-262 now read: “This model has been adapted within public and mental health research to explain the multi-level factors influencing individuals’ health behaviors and risk factors, that include: intrapersonal, interpersonal, organizational, community, policy, and societal factors [44,45]. The socio-ecological model was relevant for our study as it provided a comprehensive understanding of the factors influencing AGYW’s mental health, due to the recognition that adolescents and young people’s (AYP) social environments, including their individual experiences, family and peer relationships, and community contexts (e.g., school, and neighborhood) significantly influence their health behaviors and mental health outcomes [46,47]. We presented our findings of the key themes in the subsequent sections, organized within the different levels of the socio-ecological model (individual, interpersonal, community, provider, service, and structural-level factors).

**216: “risk factor for experiencing…”**

We have made the change to the sentence, and line 319 now reads: “Among AGYW living with HIV, HIV stigma was a common risk factor for experiencing CMDs.”

**219: “and fear of potentially transmitting…”**

We have made the change to the sentence and lines 320-322 now reads: “This included internalized stigma attributed to challenges coming to terms with their HIV status, fear of intimate relationships due to negative self-image related to their HIV-positive status, fear of rejection, and fear of potentially transmitting HIV to their sexual partners.”

**223: Maybe just me but I would want to state the service, like “as one AGYW seeking PrEP reported:”**

We have made this change throughout the results section, and line 322-327 now reads: “Among AGYW without HIV, CMD symptoms were linked to financial constraints, especially among single-parenting adolescents, and SRH issues such as unintended pregnancy, post-abortion, and post-partum complications. Other issues included infertility, sexually transmitted infections (STIs), and challenges negotiating condom use and HIV testing with sexual partners, as one AGYW seeking PrEP reported below:…”

**244: This is very interesting that AGYW are conflating any individual attention given to somehow receiving counseling…this is not commented on in discussion and I think it should be. For one, a simple change in practice is to have more individual attention and less group adherence counseling which occurs in a lot of places. Did you ask more about group versus individual services? I would look for any qualitative data about group versus individual counseling for AGYW getting HIV services and see if you see anything interesting that can be added to the paper. For sure I think group counseling may be an opportunity lost to talk to an individual AGYW about stress, anxiety, depression, GBV, poverty, etc. Generally, this is an excellent section…**

We have included a discussion of the counseling strategies in the discussion section. Lines 590-599 now read: “Few AGYW in our study had ever received mental services but considered the HIV counselling they received from HIV clinics as a form of mental health service. This could be due to the individual attention they received from HIV care providers, which they hardly received during routine services. Group-based or individual psychosocial interventions among adolescents living with or affected by HIV in SSA have reported positive emotional and behavioral outcomes [63]. While group-based interventions provide social cohesion and learning, individual models are client-centered, provide individual attention, and address individual needs [64]. Providing individual mental health screening, counseling, and referrals for AGYW within HIV clinics may present an opportunity for client-centered care while addressing CMDs.”

In the interviews, we did not ask respondents about group versus individual counseling strategies at the HIV clinics. We have included this as an area for future studies. Lines 693-700 now read: “Future studies could focus on developing and evaluating the feasibility, effectiveness, acceptability, and cost-effectiveness of tailored implementation strategies to support mental health integration for AGYW within HIV clinics. These strategies could include provider training models on mental health service delivery and AGYW-centered care, integrated mental health service delivery models including integration in stand-alone youth centers versus HIV clinics, and individualized versus group-based psychosocial interventions, specific service integration guidelines, and AGYW-friendly CMD screening tools.”

**261: Interesting that this quote is essentially the definition of resiliency. Maybe comment on that here or in discussion? Also, anything to add to discussion about stress? It seems a common complaint but also vague, any literature showing that AGYW or others use this word as an all encompassing word for a mental health concern? I heard it a lot in these kinds of settings too.**

We have included a discussion of resilience in the discussion section, and lines 576-586 now read: “Even though AGYW in this study considered mental health services to be beneficial for addressing their challenges, getting empowerment, and problem-solving, they had limited knowledge of locations where they could access these services. Other studies have shown HIV clinics to be a convenient location for adolescents on HIV care to access mental health support, since these adolescents have reported not knowing alternative locations to access psychosocial support [59]. Providing mental health services to AGYW receiving HIV services could promote their resilience through enhancing self-efficacy, motivation, and positive attitudes, essential for overcoming risk factors and continued engagement in care, contributing to positive health behaviors and outcomes [60,61]. Previous studies have highlighted the need for psychosocial support to address stigma and disclosure challenges particularly among AYP living with HIV [58].”

We have also included a discussion on the idioms of distress in the Kenyan and SSA setting in the discussion section, and lines 566-568 now read: “AGYW described experienced CMDs using idioms of distress such as ‘stress’, and ‘thinking a lot’, which have been commonly described among clients seeking care in Kenya and other SSA settings [54,55].”

**273: “delivering mental health services to AGYW than HIV providers did”. “However, HIV care providers’ lower confidence in providing mental health services was mainly due to…”**

We have revised the sentence, and lines 380-382 now read: “Generally, mental healthcare providers felt more comfortable, competent, and confident in delivering mental health services to AGYW than HIV care providers. HIV care providers’ lower confidence in providing mental health services was mainly due to limited skills.”

**277: “however, more specific screening tools …”**

We have revised lines 384-386 to now read: “Screening was primarily conducted by clinical assessment of clients’ history and symptoms; however, more specific screening tools were used for further CMD diagnosis.”

**279: internalized stigma**

We have revised lines 386-389 to now read: “Among both HIV and mental healthcare providers, mental health counseling provided to AGYW was mostly related to the reduction of internalized HIV and mental health stigma, social issues like abuse, gender-based violence, and conflicts among AGYW and their caregivers or partners…”

**285: I have never heard this ever that an HIV clinic had support groups for AGYW unless it was an outside program like DREAMS, it just doesn’t happen. I would verify this information with Kenyan colleagues or make a note anywhere you talk about support groups that they were non-governmental additional services brought by an outside group…unless he is totally making this up to make the clinic look good?**

Several AGYW living with HIV and healthcare providers in our study reported the existence of support groups in the respective HIV clinics. Support groups are used to support adherence and retention among adolescents living with HIV in many Kenyan HIV clinics and have been documented in previous studies e.g., Njuguna et al. (2020) (Reference: <https://doi.org/10.1111/tmi.13374>). Kenya’s “Adolescent’s Package of Care” (NASCOP, 2014) (Link: <https://2021-2025.state.gov/wp-content/uploads/2024/01/HCWTraining-Guideline_AdolescentpackageofcareinKen.pdf>) recommends the establishment of youth support groups as one of the components of Youth Friendly Services in HIV clinics.

**297: You just wrote that AGYW get referred. So is this counseling done by the clinic where the AGYW presented or at the referral site? Need to clarify.**

We have clarified the sentence, and lines 407-409 now read: “Within HIV clinics, AGYW counseling and follow-up was focused on information and adherence support for HIV prevention and treatment, and linkages to psychosocial support groups for AGYW living with HIV.”

**306: This is another quote that needs verification I believe. You may just want to find out what % of AGYW (or any patient) is physically escorted. This is so uncommon in these settings and this nurse may not be entirely truthful about all AGYW being physically escorted, this is so uncommon in a very busy clinic that staff can escort patients. Or explain if we are talking about a different department at a hospital??**

We have clarified the sentence to provide context and replaced the word ‘all’ with ‘most’ since at least four providers in three facilities mentioned that they provided escorted referrals to adolescent clients- this could be within the same HIV clinic building or to a separate mental health department.

Lines 411-414 now read: “Most AGYW referrals were done through ‘referral by escort’, whereby HIV care providers physically escorted AGYW to relevant providers, e.g., psychologists located within HIV clinics, or externally to mental health clinics located within the facility.”

**310: Consider ‘access’ instead of delivery and ‘by AGYW’; check abstract an intro to see if factors influencing access to services is coming through strongly enough …**

We have made changes to include both ‘access’ and ‘delivery’ factors from the abstract, introduction, and results sections.

In the abstract, lines 56-57 now read: “AGYW reported a high need for mental health services but described a systemic lack of access. Convenience and positive experiences with providers facilitated AGYW’s access to services.” and lines 67-68 now read: “Integrated service delivery within HIV clinics could improve AGYW’s access to mental health services.”

In the introduction, lines 95-96 now read: “Integrating mental health services within HIV clinics could improve access to mental healthcare among vulnerable AGYW populations [8].”

Lines 111-114 now read: “Numerous barriers hinder mental health service access and delivery among adolescents receiving HIV services in SSA, including stigma, shortage of trained mental healthcare providers, poor treatment and referral pathways, and a lack of routine screening for CMDs [13,15,16].”

In the results, line 422 now reads: “Factors influencing mental health service access and delivery among AGYW.”

**339: consider, “Conversely, mental healthcare providers…”**

We have made this change, and lines 454-455 now read: “Conversely, mental healthcare providers had adequate training and considered mental health services as their core evaluable role.”

**344: rewrite for better clarity**

We have rewritten the sentence in lines 458-461, which now reads: “HIV care providers also reported difficulties when counseling AGYW with persistently high viral loads, noting that these AGYW often became angry with providers, refused to answer adherence-related questions, and defaulted on their monthly clinic visits.”

**349: Don’t need however. What does “difficulty of AGYW gaining insight” mean?**

We have clarified this sentence and lines 465-468 now read: “Among mental healthcare providers, key challenges included difficulty of AGYW gaining insight (i.e., ability to recognize and accept their CMD symptoms), AGYW declining mental health services and medication, mental health stigma, and lack of treatment support from caregivers and partners.”

**350: mental health stigma?**

We have made this change and lines 465-468 now read: “Among mental healthcare providers, key challenges included difficulty of AGYW gaining insight (i.e., ability to recognize and accept their CMD symptoms), AGYW declining mental health services and medication, mental health stigma, and lack of treatment support from caregivers and partners.”

**353-359: I don’t see this quote as showing that some people think mental health care providers behave like their clients, I would delete that conclusion. They are just saying that providers may be afraid of MH patients because they could be aggressive or harm them.**

We have revised the sentence and lines 468-471 now read: “Mental healthcare providers also reported stigma and discrimination from other providers, especially those in non-psychiatric departments, who avoided interacting with clients with mental health disorders and used stigmatizing words like ‘Mwendawazimu’ (Kiswahili- mad person) to refer to clients…”

**366: “since adolescents had more routine HIV care parameters” what does this mean? Rewrite these sentences here because it is important to note clearly why providers thought it was different to work with adolescents and why exactly.**

We have clarified this sentence, and lines 484-487 now read: “Further concerns included the lengthy time needed to provide HIV services to AGYW since providers had to complete more documentation for AGYW aged up to 25 years who were in HIV care compared to adults.”

**369: “screening into HIV clinics” or into HIV service delivery**

We have revised the sentence in line 487-490 to now read: “Providers also reported concerns about competing roles, high workload, time constraints related to high client volumes within public HIV clinics, and a lack of clear guidelines and practical tools to support HIV care providers on integrating mental health screening into HIV service delivery.”

**389: Benefit of integrating …?**

We have revised the heading in line 511 which now reads: “Benefits of integrating mental health services within HIV clinics”

**390-396: needs rewrite for more clarity**

We have rewritten the section in lines 513-521 which now read: “Both AGYW and providers supported integration of mental health services within HIV clinics, acknowledging the need for these services among AGYW. Providers specifically emphasized the need for integration due to the high vulnerability of AGYW to CMDs, particularly among those receiving HIV treatment. This concern was attributed to the high numbers of AGYW who are currently referred to psychiatric clinics, and those attending both HIV and psychiatric clinics simultaneously. However, both AGYW and providers reported a gap in the integration of mental health services within HIV services for AGYW, as one HIV counselling provider reported below:…”

**409: CMD symptoms, substance use relapses, …**

We have revised the sentence in lines 533-535 which now reads: “AGYW and providers also expected integration to result in overall improved quality of care, hence yielding better mental health and HIV outcomes, for instance, reduced CMD symptoms and substance use relapses, improved HIV prevention and treatment adherence and persistence, and viral suppression…”

**415-431: See notes above about individual versus group counseling, did this come up in the interviews re. how to integrate MH services**

In the interviews, we did not ask respondents about group versus individual counseling strategies at the HIV clinics. We have included this in the discussion section and listed it as a gap for future studies on the feasibility of integration.

Lines 593-599 now read: “Group-based or individual psychosocial interventions among adolescents living with or affected by HIV in SSA have reported positive emotional and behavioral outcomes [63]. While group-based interventions provide social cohesion and learning, individual models are client-centered, provide individual attention, and address individual needs [64]. Providing individual mental health screening, counseling, and referrals for AGYW within HIV clinics presents an opportunity for client-centered care while addressing CMDs.”

Lines 693-700 now read: “Future studies could focus on developing and evaluating the feasibility, effectiveness, acceptability, and cost-effectiveness of tailored implementation strategies to support mental health integration for AGYW within HIV clinics. These strategies could include provider training models on mental health service delivery and AGYW-centered care, integrated mental healthcare delivery models including integration in stand-alone youth centers versus HIV clinics, and individualized versus group-based psychosocial interventions, specific service integration guidelines, and AGYW-friendly CMD screening tools.”

**Discussion**

**436: “contrasting service access and delivery gaps among AGYW and providers”. What does this mean? Can be stated in plain language like differences of opinion or something like that.**

We have revised the sentence in lines 561-564, which now reads: “There is a high need for and acceptability of mental health service delivery within HIV clinics, but a contrastingly low access to these services among AGYW, and notable service delivery gaps among providers.”

**446-450: This jumps out early in discussion, esp. after just reading those findings the page before. Suggest moving this to join with other service integration language later in Discussion.**

We have moved this sentence into the discussion on integration, and lines 670-675 now read: “Overall, integration of mental health within HIV services was highly acceptable among AGYW and providers for addressing CMDs among AGYW receiving HIV services. Integration was expected to potentially yield multiple benefits such as reduced referrals to over-burdened mental healthcare providers, efficiency and convenience of providing services ‘under one roof’, timely intervention for CMDs, improved quality of care, and better mental health and HIV outcomes.”

**473-476: why are they more challenging? I’m still not seeing why. Cite any literature where providers consider serving adolescents as like serving children, they are looked down on as a population to serve.**

We have included a discussion of the concerns and perceived challenges among providers with providing services to adolescents, and lines 637-656 now read: “In our study, providers’ reluctance to provide services to AGYW due to perceptions that AGYW were ‘difficult clients’ hindered mental health service delivery to AGYW. HIV and mental healthcare providers also perceived delivering mental health services to AGYW to be more challenging and time-consuming compared to other populations, and as ‘extra work’. A recent study among HIV care providers in Kenya, South Africa, and Zimbabwe reported challenges in delivering PrEP services to adolescent girls younger than 18 years, whom providers described as ‘troublesome’, ‘not listening’, and ‘dishonest’ [69]. Other studies have reported beliefs among providers regarding the perceived difficulty of providing care to adolescents with CMDs [53], and the burden from additional screening tools [7]. Studies in SSA have also reported biases among SRH and HIV care providers regarding providing contraception and HIV prevention to AGYW due to cultural stigma towards pre-marital sexual activity among youth [70,71]. In Kenya, low confidence to deliver HIV services to adolescents has been attributed to insufficient provider training on adolescent-friendly HIV care, while access to trained providers and youth-friendly services have been linked to better HIV outcomes [72,73]. In our study, HIV care providers employed youth-friendly strategies like ‘referral by escort’ but lacked specific skills for addressing CMDs and for AGYW-friendly communication and counseling. These findings could explain the providers’ reluctance to deliver mental health services to AGYW, highlighting the need to prioritize the provision of regular provider training on mental health services and adolescent-friendly care.”

**Please see attached Word document with detailed review of paper. Overall: it seems that you have chosen to endorse integration over a stand alone youth center, as mentioned by the providers and which is in fact is a very common way to deal with the very real difficulties in integrating adolescent care into an adult setting. This again is a priori coding and should be stated as such, that you were interested in finding out AGYW and providers view on integration…but take a look to see if you can also mention that some providers support youth centers as an alternative and that perhaps future studies should compare and contrast the two models for adolescent mental health, including cost, to determine which model leads to better outcomes.**

We have included this discussion as a potential area to explore in future studies. Lines 693-700 now read: “Future studies could focus on developing and evaluating the feasibility, effectiveness, acceptability, and cost-effectiveness of tailored implementation strategies to support mental health integration for AGYW within HIV clinics. These strategies could include provider training models on mental health service delivery and AGYW-centered care, integrated mental health service delivery models including integration in stand-alone youth centers versus HIV clinics, and individualized versus group-based psychosocial interventions, specific service integration guidelines, and AGYW-friendly CMD screening tools.”
