# Supplementary material for: Mental health service delivery among adolescent girls and young women (AGYW) seeking HIV prevention and treatment services in central Kenya: A qualitative study of AGYW and healthcare providers’ perceptions
Source: PLoS One. 2025 Dec 5;20(12):e0337795. doi: 10.1371/journal.pone.0337795 (PMC12680144; doi:10.1371/journal.pone.0337795)
Supplement: Attachment — Submitted filename: Review Notes.docx [file pone.0337795.s008.docx]

**Review Notes:**

Overall: This is much improved from the original. In the methods I would say more about the items, scales or questions in the SRQ-20. A major limitation is that the MH services in these settings largely don’t exist so the interviews are partly based on a hypothetical, but this is not addressed in discussion. Also, the discussion doesn’t really address whether HIV positive or HIV negative (seeking prevention services) need different MH services or perhaps if HIV positive AGYW should at least at the beginning, be prioritized.

Abstract:

34: consider AGYW instead of ‘they’

39: do you need apostrophe at the end?

43: consider revising to make clear this is AGYW only; perhaps revise to include providers somehow

50: consider “due” multi-level risk factors and also “financial problems”

51: you refer to need throughout, this could be considered “demand” for mental health services

Introduction:

73: “poor adherence” leading to uncontrolled viral load and poor treatment outcomes (I believe this should be spelled out more since it is a serious consequence

91-93: consider moving to earlier in introduction (line 82)

108-109: “explored mental health service delivery” this is partially misleading the reader, since you clearly state that such services don’t exist per se beyond adherence counseling; you are more exploring the potential that exists

110: take out apostrophe after Central

Methods:

Even though you don’t present findings on the SRQ-20 (you may want to consider including these data to make the paper more robust, though I realize it is a qualitative report) it would be good to get some inkling of the main domains in this tool, what is measured, etc.

122: add “study-related” screening and enrollment, I read it like they getting services

126: where did screening occur? In the clinic? Was it private?

129: eligible for in depth interviews

131: consent to participation

132: start new sentence after the age groups are noted

136: consider new paragraph for methods related to providers

138: this is a hold over from previous draft; I am still confused that you state earlier you are working in 2 clinics but then the providers are from a much larger region; perhaps you can add a statement that providers were not just recruited from the two clinics where the AGYW were seeking HIV services but from a broader area (and why you recruited that way)

Data collection explanation is very good

169: it isn’t clear if the social-ecological model was used for both sets of IDIs? For the providers as well?

Results:

196: add range for years of service for providers

198: consider replacing “broadly” with often

206: “were more likely”

216: “for experiencing CMDs”

223: remove apostrophe after condom use

228: this would be more clear to me if you talk about AGYW experiences of mistreatment due to HIV-related (or even HIV status related…this does not get explicitly stated in the paper, it is assumed we know the stigma is due to HIV status)

245: the fact that less than half of the AGYW reported receiving mental health services is a limitation of this study; they do not have the experience from which to draw their opinions and ideas, which is central to qualitative work, add to limitations

248-255: this whole section is making conclusions with no quote, perhaps add a quote here

259-260: This sentence is really unclear, needs rewriting

277: “were also used”

279: “related to addressing HIV and mental health stigma”

296: to rule out mental health “or substance use” issues

297-299: this sentence seems redundant to what has already been stated, could be deleted

341-348: this whole section is making conclusions with no quote, perhaps add a quote here

344: “perceived easily got angry at providers” needs rewriting

349: consider “conversely” or another word instead of however

350: “mental health stigma”

351-352: add “observing” stigma and discrimination or it sounds like providers themselves experienced it

363: facilities seems like a typo, should be “consultations” or similar

365-368: I’m seeing what you are trying to say but it isn’t well written, consider revising for clarity. It is an important point.

383: no apostrophe needed after policies

393: what does “related to” mean here? This sentence is unclear.

395: disparity for which group, AGYW?

Discussion:

What do you think about the different findings for HIV positive versus HIV negative AGYW? What are the implications?

434: I would say various CMD symptoms or something like that because you did not present data on the number of symptoms

436: what does contrasting mean here? Disagreement?

445: ability and confidence

461: “likely explains” I think

467-476: In SSA generally there is a widespread belief that adolescents should not be afforded the same access to services and attention that adults get and that they don’t have the rights an adult would have, and this impacts health care delivery to adolescents. Leadership at MOH and elsewhere has to set the tone that adolescents deserve good health care just like an adult. Did this come up in your interviews? You may want to see if you can find citations about this phenomenon and add a sentence or two, but it could underly providers’ resentment regarding service delivery to adolescents and spending lots of time and effort to provide services to an individual who is not even an adult (a child in their eyes).

483: I would replace outcomes with “generally”, providers don’t promote outcomes they promote mental health

509: developing and evaluating
