# Supplementary material for: Mental health service delivery among adolescent girls and young women (AGYW) seeking HIV prevention and treatment services in central Kenya: A qualitative study of AGYW and healthcare providers’ perceptions
Source: PLoS One. 2025 Dec 5;20(12):e0337795. doi: 10.1371/journal.pone.0337795 (PMC12680144; doi:10.1371/journal.pone.0337795)
Supplement: Attachment — Submitted filename: Response_to_Reviewers_auresp_2.docx [file pone.0337795.s010.docx]

14^th^ August 2025

The Academic Editor

PLOS ONE

Dear Dr. Chen,

RE: Response to Manuscript ID PONE-D-24-60333R1

We thank the reviewers for your review of our manuscript entitled, “**Mental health service delivery among adolescent girls and young women (AGYW) seeking HIV prevention and treatment services in central Kenya: a qualitative study of AGYW and healthcare providers’ perceptions**”, and the opportunity to resubmit a revised version. We appreciate your and the reviewers’ valuable feedback, and we are pleased to incorporate these recommendations. We are resubmitting the revised manuscript that addresses all suggestions. We noticed that we had already addressed some of the comments in the previous revision submitted on 30^th^ March 2025 (Manuscript Number: PONE-D-24-60333R1), which we have indicated in our responses here. Below is an updated point-by-point response to the comments along with a description of the changes that have been made to address the comments. Page and line numbers indicated below refer to the clean version of the manuscript. The reviewers’ comments are included in bold text, and our responses are standard font. We appreciate, in advance, your time and consideration of this revised manuscript for publication in PLOS ONE.

Sincerely,

Jennifer Velloza, PhD, MPH (on behalf of all authors)

University of California, San Francisco (UCSF)

**Reviewer #1 Comments:**

**Thank you for the thorough responses to my reviewer suggestions. The manuscript reads clearly and concisely, and will be of great value to the field.**

We thank the reviewer for this positive feedback.

**Reviewer #2 Comments:**

**A much better draft, well done. Please see my notes for a final revision. Overall: This is much improved from the original.**

We thank the reviewer for this positive feedback.

**In the methods I would say more about the items, scales or questions in the SRQ-20.**

For this study, our analysis focused on the qualitative findings since the SRQ-20-item screening tool was only used for eligibility assessment for interview participation. We provided more detail on the SRQ-20 in our primary quantitative paper: Concepcion et al., (2023). Higher rates of mental health screening of adolescents recorded after provider training using simulated patients in a Kenyan HIV clinic: results of a pilot study. doi: 10.3389/fpubh.2023.1209525, which we have cited in line 128 of the current manuscript. We have also included a copy of the SRQ-20-item questionnaire that we used for screening AGYW as a supporting file which is cited in line 160 for further reference.

**A major limitation is that the MH services in these settings largely don’t exist, so the interviews are partly based on a hypothetical, but this is not addressed in discussion.**

We have included a discussion on AGYW’s limited experiences with receiving mental health services in the discussion section, and lines 653-657 now read: “The study had some limitations. First, we used a small sample size, limiting the generalizability of findings to the general population. Additionally, AGYW had limited experiences with receiving mental health services and were unfamiliar with some of the concepts around integrated service delivery that were discussed, which may have impacted some of their responses about CMD symptoms and mental health services.”

**Also, the discussion doesn’t really address whether HIV positive or HIV negative (seeking prevention services) need different MH services or perhaps if HIV positive AGYW should at least at the beginning, be prioritized.**

From our findings, mental health services are important and should be prioritized for all AGYW receiving HIV services regardless of their HIV status. We have included a discussion on the prioritization of MH services for AGYW in lines 541-543 which now read: “Prioritizing routine screening for CMDs and psychosocial interventions that address key CMD risk factors and specific needs of AGYW is essential; and should be integrated in status-neutral HIV service delivery for both AGYW receiving HIV treatment and prevention services [59].”

Abstract:

**34: consider AGYW instead of ‘they’**

We previously addressed this comment and made changes to line 34-35 which now reads: “However, mental health is under-prioritized within HIV interventions targeting AGYW.”

**39: do you need apostrophe at the end?**

We previously addressed this comment and removed the apostrophe in lines 38-39 which now read: “Between 16th February and 14th June 2021, we conducted in-depth interviews with AGYW receiving HIV services and healthcare providers from eight clinics in Central Kenya.”

**43: consider revising to make clear this is AGYW only; perhaps revise to include providers somehow**

We have revised the sentence to specify experiences of CMDs among AGYW and included providers, and lines 42-44 now read: “Interviews explored AGYW’s experiences with CMDs and factors influencing mental health service delivery by providers within HIV clinics.”

**50: consider “due” multi-level risk factors and also “financial problems”**

We have revised the sentence and lines 49-50 now read: “AGYW described experiences of CMDs due to multi-level risk factors, including HIV stigma, financial problems, and relationship challenges.”

**51: you refer to need throughout, this could be considered “demand” for mental health services**

We have revised this sentence and throughout the manuscript and lines 50-51 now read: “AGYW reported a high demand for mental health services but described a systemic lack of access.”

Introduction:

**73: “poor adherence” leading to uncontrolled viral load and poor treatment outcomes (I believe this should be spelled out more since it is a serious consequence)**

We have made the suggested changes to lines 81-84 which now read: “CMDs undermine HIV outcomes among AGYW by contributing to low service uptake and retention, poor adherence leading to uncontrolled viral load and poor treatment outcomes, limited engagement and persistence in care, and increased sexual risk behaviors, exacerbating AGYW’s risk of HIV acquisition and transmission.”

**91-93: consider moving to earlier in introduction (line 82)**

We have made changes and moved this sentence to earlier in the introduction in lines 77-80 which now read: “Numerous barriers hinder mental health service access and delivery among adolescents receiving HIV services in SSA, including stigma, shortage of trained mental healthcare providers, poor treatment and referral pathways, and a lack of routine screening for CMDs [13–15].”

**108-109: “explored mental health service delivery” this is partially misleading the reader, since you clearly state that such services don’t exist per se beyond adherence counseling; you are more exploring the potential that exists**

We have made changes to the sentence in lines 117-119 which now read: “We conducted a qualitative study to understand the perceptions around potential expansion of mental health service access and delivery within HIV clinics among AGYW receiving HIV services and healthcare providers in Central Kenya.”

**110: take out apostrophe after Central**

We have made changes to the sentence in lines 117-119 which now read: “We conducted a qualitative study to understand the perceptions around potential expansion of mental health service access and delivery within HIV clinics among AGYW receiving HIV services and healthcare providers in Central Kenya.”

Methods:

**Even though you don’t present findings on the SRQ-20 (you may want to consider including these data to make the paper more robust, though I realize it is a qualitative report) it would be good to get some inkling of the main domains in this tool, what is measured, etc.**

For this study, our analysis focused on the qualitative findings since the SRQ-20-item screening tool was only used for eligibility assessment for interview participation. We provided more detail on the SRQ-20 in our primary quantitative paper: Concepcion et al., (2023). Higher rates of mental health screening of adolescents recorded after provider training using simulated patients in a Kenyan HIV clinic: results of a pilot study. doi: 10.3389/fpubh.2023.1209525, which we have cited in line 128 of the current manuscript. We have also included a copy of the SRQ-20-item questionnaire that we used for screening AGYW as a supporting file which is cited in line 160 for further reference.

**122: add “study-related” screening and enrollment, I read it like they getting services**

We have made this change and lines 149-150 now read: “AGYW were referred for study-related screening and enrollment for the study by HIV care providers from Thika sub-County Hospital and the PHRD clinic.”

**126: where did screening occur? In the clinic? Was it private?**

Screening occurred in a private clinic room at the PHRD clinic. We have made this change, and lines 158-161 now read: “Once the AGYW arrived at the PHRD clinic, they were screened for study participation by a study psychosocial counselor in a private clinic room using the Self Reporting Questionnaire 20-item (SRQ-20) mental health screening tool [38] (Supporting File 1), to assess for elevated symptoms of CMDs.”

**129: eligible for in depth interviews**

We have made this change and lines 161-162 now read: “AGYW with mild-to-moderate CMD symptoms (determined by a score of 7-14 on the SRQ-20 screening tool), were eligible for in-depth interviews.”

**131: consent to participation**

We have made this change and lines 162-165 now read: “AGYW with scores above 14, indicating severe CMD symptoms, were excluded from interviews due to potential impacts on the capacity to consent to study participation, and risk of harm related to emotional distress from discussing sensitive topics or traumatic experiences [39].”

**132: start new sentence after the age groups are noted**

We have made this change to start a new sentence, and lines 174-177 now read: “We included AGYW in different age groups (16-18, 19-22, and 23-25). These AGYW were receiving a variety of HIV prevention and treatment services including: PrEP, HIV testing services (HTS), post-exposure prophylaxis (PEP), and antiretroviral therapy (ART).”

**136: consider new paragraph for methods related to providers**

We have started a new paragraph for methods related to providers in lines 167-171 and lines 178-182.

**138: this is a hold over from previous draft; I am still confused that you state earlier you are working in 2 clinics but then the providers are from a much larger region; perhaps you can add a statement that providers were not just recruited from the two clinics where the AGYW were seeking HIV services but from a broader area (and why you recruited that way)**

We have made changes to clarify the study recruitment sites for AGYW and healthcare providers at the beginning of the participant recruitment section and lines 144-148 now read: “AGYW were recruited from Thika sub-County Hospital and the PHRD clinic. Healthcare providers were recruited from Thika sub-County Hospital and six surrounding facilities to capture broader perspectives on mental health service delivery to AGYW within the larger catchment area that provides referrals to the primary study site.”

We also included the rationale for involving additional facilities earlier in the study setting and population section in lines 136-141 which now read: “We also included seven surrounding healthcare facilities to increase the diversity of the study population. These included Partners in Health and Research Development (PHRD), a clinical research site that implements HIV prevention and treatment trials among diverse populations including AGYW, Makongeni dispensary (Level 2), Kiandutu health centre (Level 3), Ruiru sub-County hospital (Level 4), Kiambu County referral hospital (Level 5), Mathari National referral hospital (Level 6), and Kenyatta National hospital (Level 6).”

**Data collection explanation is very good**

We thank the reviewer for this positive feedback.

**169: it isn’t clear if the social-ecological model was used for both sets of IDIs? For the providers as well?**

Yes, we used the socio-ecological model for both sets of IDIs. We have clarified this in the data management section and lines 219-222 now read: “We used the socio-ecological model to categorize themes around the individual, interpersonal, community, and structural factors influencing mental health service delivery, access, and utilization among AGYW receiving HIV services and providers delivering HIV and mental health services in the respective facilities.”

Results:

**196: add range for years of service for providers**

We have made the suggested change and lines 258-261 now read: “Providers had a median age of 37 years (IQR: 30-44). The sample included 70% (n=7) females, with a median of 7 years of experience (IQR: 5-17) in HIV or mental health service delivery; their educational backgrounds included diploma (n=6), higher diploma (n=2) and degree (n=2).”

**198: consider replacing “broadly” with often**

We have made this change and lines 266-268 now read: “AGYW often described any experiences of CMDs as ‘stress’ and used words in English, Kiswahili, or ‘Sheng’ (an urban slang popular among Kenyan youth) languages to describe their mental health symptoms.”

**206: “were more likely”**

We have made this change and lines 273-275 now read: “Symptoms like self-isolation, changes in sleep and appetite, and drug and alcohol use were more likely to be reported among AGYW with lower SRQ-20 screening scores (7-10).”

**216: “for experiencing CMDs”**

We previously addressed this comment and made changes to line 285 which now reads: “Among AGYW living with HIV, HIV stigma was a common risk factor for experiencing CMDs.”

**223: remove apostrophe after condom use**

We previously addressed this comment and removed the apostrophe in lines 291-293 which now read: “Other issues included infertility, sexually transmitted infections (STIs), and challenges negotiating condom use and HIV testing with sexual partners, as one AGYW seeking PrEP reported below:”

**228: this would be more clear to me if you talk about AGYW experiences of mistreatment due to HIV-related stigma (or even HIV status related…this does not get explicitly stated in the paper, it is assumed we know the stigma is due to HIV status)**

We have clarified the results to specify HIV-related stigma as a risk factor for CMDs. Lines 298-303 now read: “Among AGYW living with HIV, CMD symptoms were also attributed to experiences of HIV-related stigma from discrimination, abuse, and withholding of emotional, material, and treatment support by peers, partners, and caregivers due to AGYW’s HIV status. Furthermore, perceived and anticipated HIV-related stigma was related to lack of privacy in HIV clinics, schools, and the community, which presented a risk of inadvertent HIV status disclosure and affected discreetness in taking medication.”

**245: the fact that less than half of the AGYW reported receiving mental health services is a limitation of this study; they do not have the experience from which to draw their opinions and ideas, which is central to qualitative work, add to limitations**

We have included a discussion on AGYW’s limited experiences with receiving mental health services in the discussion section, and lines 653-657 now read: “The study had some limitations. First, we used a small sample size, limiting the generalizability of findings to the general population. Additionally, AGYW had limited experiences with receiving mental health services and were unfamiliar with some of the concepts around integrated service delivery that were discussed, which may have impacted some of their responses about CMD symptoms and mental health services.”

**248-255: this whole section is making conclusions with no quote, perhaps add a quote here**

We have included a quote to support the results as suggested. Lines 319-328 now read: “However, we found that most AGYW who reported receiving mental health services in HIV clinics had only received HIV adherence counseling and follow-up specific to HIV and STI prevention and treatment services (e.g., HIV testing, HIV status disclosure, and medication adherence) from HIV counseling providers, as one AGYW seeking PEP described below:

Okay, what she [provider] did is the counseling: she told me about how I can protect myself, about how I can also use PrEP, she also told me about how I am going to take my medication (PEP). Yeah, she just told me about preventive measures; about how I can also cope with people who are HIV-positive [living with HIV] and not get infected. (AGYW, Age 24, Seeking HTS/PEP, SRQ-20 Screening Score 11).”

**259-260: This sentence is really unclear, needs rewriting**

We have revised the sentence in lines 337-340 which now read: “AGYW said that receiving mental health support from HIV care providers would be beneficial for getting relief from mental health burdens, empowering them, and helping them potentially find solutions to their problems, as one AGYW seeking HTS and PEP described below:”

**277: “were also used”**

We have made this change and lines 356-358 now read: “Screening was primarily conducted by clinical assessment of clients’ history and symptoms. However, more specific screening tools were also used for further CMD diagnosis.”

**279: “related to addressing HIV and mental health stigma”**

We have made this change and lines 358-362 now read: “Among both HIV and mental healthcare providers, mental health counseling provided to AGYW was mostly related to addressing internalized HIV and mental health stigma, social issues like abuse, gender-based violence, and conflicts among AGYW and their caregivers or partners, SRH issues such as unintended pregnancies, and contraceptive use, substance use addiction, grief, and life skills.”

**296: to rule out mental health “or substance use” issues**

We have made this change and lines 376-378 now read: “These tools were integrated into the Electronic Medical Records (EMR) system, for screening clients with high viral load, to rule out mental health or substance use issues as possible adherence barriers.”

**297-299: this sentence seems redundant to what has already been stated, could be deleted**

We have deleted the sentence previously in lines 378-381.

**341-348: this whole section is making conclusions with no quote, perhaps add a quote here**

We have included a quote in this section and lines 426-433 now read:

HIV care providers also reported difficulties when counseling AGYW with persistently high viral loads, noting that these AGYW often became angry with providers, refused to answer adherence-related questions, and defaulted on their monthly clinic visits, as one provider described below:

The challenge comes with the high viral load ones [AGYW] where you have now to see them every month and ask, “Why, why, why is your viral load up?” It gets to a point when they start getting angry at you. So, they get angry at you and then they start just keeping quiet at you, so it is you now to decide how you will probe and get to the bottom of the issues…So, like those ones now…I can refer very fast. (Male Clinical Officer, HIV Clinic).

**344: “perceived easily got angry at providers” needs rewriting**

We previously addressed this comment and made changes in lines 426-428 which now read: “HIV care providers also reported difficulties when counseling AGYW with persistently high viral loads, noting that these AGYW often became angry with providers, refused to answer adherence-related questions, and defaulted on their monthly clinic visits.”

**349: consider “conversely” or another word instead of however**

We have made this change and lines 438-441 now read: “Conversely, among mental healthcare providers, key challenges included difficulty of AGYW gaining insight (i.e., ability to recognize and accept their CMD symptoms), AGYW declining mental health services and medication, mental health stigma, and lack of treatment support from caregivers and partners.”

**350: “mental health stigma”**

We previously addressed this comment and made this change in lines 438-441 which read: “Conversely, among mental healthcare providers, key challenges included difficulty of AGYW gaining insight (i.e., ability to recognize and accept their CMD symptoms), AGYW declining mental health services and medication, mental health stigma, and lack of treatment support from caregivers and partners.”

**351-352: add “observing” stigma and discrimination or it sounds like providers themselves experienced it**

We have made this change and lines 441-444 now read: “Mental healthcare providers also reported observing stigma and discrimination from other providers, especially those in non-psychiatric departments, who avoided interacting with clients with mental health disorders and used stigmatizing words like ‘Mwendawazimu’ (Kiswahili- mad person) to refer to clients,”

**363: facilities seems like a typo, should be “consultations” or similar**

We have made this change and lines 451-453 now read: “Among HIV care providers, structural factors affecting mental health service delivery to AGYW included a lack of funds for provider training and inadequate rooms for adolescent-friendly consultations, which resulted in a lack of privacy for AGYW.”

**365-368: I’m seeing what you are trying to say but it isn’t well written, consider revising for clarity. It is an important point.**

We have made this change and lines 453-455 now read: “HIV care providers also experienced difficulties escorting AGYW to and from mental health clinics for their referrals, due to challenges around lack of time, busy clinic workloads, and staffing shortages.”

**383: no apostrophe needed after policies**

We have made this change and lines 474-476 now read: “Limited resource allocation to mental health units within public health facilities, shortage of in-patient facilities, and restrictive facility policies further limited mental health services delivery to AGYW,”

**393: what does “related to” mean here? This sentence is unclear.**

We previously addressed this comment and made this change in lines 484-488 which now read: “Providers specifically emphasized the need for integration due to the high vulnerability of AGYW to CMDs, particularly among those receiving HIV treatment. This concern was attributed to the high numbers of AGYW who are currently referred to psychiatric clinics and those attending both HIV and psychiatric clinics simultaneously.”

**395: disparity for which group, AGYW?**

We have clarified this sentence, and lines 488-489 now read: “However, both AGYW and providers reported a lack of integration of mental health services within HIV services for AGYW, as one HIV counselling provider reported below:”

Discussion:

**What do you think about the different findings for HIV positive versus HIV negative AGYW? What are the implications?**

From our findings, mental health services are important and should be prioritized for all AGYW receiving HIV services regardless of their HIV status. We have included a discussion on the prioritization of MH services for AGYW in lines 541-543 which now read: “Prioritizing routine screening for CMDs and psychosocial interventions that address key CMD risk factors and specific needs of AGYW is essential; and should be integrated in status-neutral HIV service delivery for both AGYW receiving HIV treatment and prevention services [59].”

**434: I would say various CMD symptoms or something like that because you did not present data on the number of symptoms**

We have made this change, and lines 526-528 now read: “Our findings reveal that AGYW receiving HIV treatment and prevention services experience various CMD symptoms and multi-level risk factors for CMDs but have limited access to mental health services beyond the standard of care HIV and STI counseling.”

**436: what does contrasting mean here? Disagreement?**

We have clarified this sentence, and lines 528-530 now read: “There is a high demand for and acceptability of mental health service delivery within HIV clinics; however, access to these services is notably low among AGYW, and gaps in service delivery exist among providers.”

**445: ability and confidence**

We previously addressed this comment and made this change in lines 583-585 which now read: “Among providers, individual and structural-level factors, including mental health training, finances, staffing, infrastructure, referral systems, and mental health guidelines, influenced the ability and confidence to deliver mental health services.”

**461: “likely explains” I think**

We have made this change, and lines 576-579 now read: “Our findings indicate that the desire to avoid anxiety, stigma, and judgment from providers likely influenced AGYW’s preference for receiving services from familiar, welcoming providers and attending few service delivery points.”

**467-476: In SSA generally there is a widespread belief that adolescents should not be afforded the same access to services and attention that adults get and that they don’t have the rights an adult would have, and this impacts health care delivery to adolescents. Leadership at MOH and elsewhere has to set the tone that adolescents deserve good health care just like an adult. Did this come up in your interviews? You may want to see if you can find citations about this phenomenon and add a sentence or two, but it could underly providers’ resentment regarding service delivery to adolescents and spending lots of time and effort to provide services to an individual who is not even an adult (a child in their eyes).**

We did not hear this in our interviews since most providers recognized the importance of delivering mental health services to AGYW but were limited by knowledge, skills and other structural barriers to providing care. However, we found negative attitudes among some providers mostly related to the perceptions of AGYW being ‘difficult’ clients, which we highlighted as a barrier in the results section in lines 434-437.

We previously addressed this comment and included a discussion on the reluctance of providers in SSA to provide mental health services to AGYW in the discussion section in lines 590-607 which now reads: “In our study, some providers were reluctant to provide services to AGYW due to perceptions that AGYW were ‘difficult clients’, which hindered mental health service delivery to AGYW. HIV and mental healthcare providers also perceived delivering mental health services to AGYW to be more challenging and time-consuming compared to other populations, and as ‘extra work’. A recent study among HIV care providers in Kenya, South Africa, and Zimbabwe reported challenges in delivering PrEP services to adolescent girls younger than 18 years, whom providers described as ‘troublesome’, ‘not listening’, and ‘dishonest’ [69]. Other studies have reported beliefs among providers regarding the perceived difficulty of providing care to adolescents with CMDs [53], and burden from additional screening tools [7]. Studies in SSA have also reported biases among SRH and HIV care providers regarding providing contraception and HIV prevention to AGYW due to cultural stigma towards pre-marital sex among youth [70,71]. In Kenya, low confidence to deliver HIV services to adolescents has been attributed to insufficient provider training on adolescent-friendly HIV care, while access to trained providers and youth-friendly services have been linked to better HIV outcomes [72,73]. In our study, although HIV care providers employed youth-friendly strategies like ‘referral by escort’, they lacked specific skills to address CMDs and for AGYW-friendly communication and counseling, which likely explains their reluctance to deliver mental health services to AGYW. These findings highlight the need to prioritize regular provider training on mental health service delivery and adolescent-friendly care.”

**483: I would replace outcomes with “generally”, providers don’t promote outcomes they promote mental health**

We have made this change, and lines 612-614 now read: “Our findings are consistent with other studies globally, which have shown that mental health interventions among AGYW largely focus on HIV treatment adherence and SRH outcomes but less on promoting mental health [8].”

**509: developing and evaluating**

We previously addressed this comment and made this change in lines 643-645, which now read: “Future studies could focus on developing and evaluating the feasibility, effectiveness, acceptability, and cost-effectiveness of tailored implementation strategies to support mental health integration for AGYW within HIV clinics.”
